# Evolutionary and genomic comparisons of hybrid uninucleate and nonhybrid *Rhizoctonia* fungi

**DOI:** 10.1038/s42003-021-01724-y

**Published:** 2021-02-15

**Authors:** Cheng Li, Zejian Guo, Shanyue Zhou, Qingyue Han, Manman Zhang, Youliang Peng, Tom Hsiang, Xujun Chen

**Affiliations:** 1 Key Laboratory of Pest Monitoring and Green Management, MOA; Joint Laboratory for International Cooperation in Crop Molecular Breeding; Department of Plant Pathology, China Agricultural University, Beijing, China; 2 College of Plant Health and Medicine, Qingdao Agricultural University, Qingdao, Shandong China; 3 Environmental Sciences, University of Guelph, Guelph, Ontario Canada

**Keywords:** Fungal genomics, Fungal evolution, Pathogens

## Abstract

The basidiomycetous fungal genus, *Rhizoctonia*, can cause severe damage to many plants and is composed of multinucleate, binucleate, and uninucleate species differing in pathogenicity. Here we generated chromosome-scale genome assemblies of the three nuclear types of *Rhizoctonia* isolates. The genomic comparisons revealed that the uninucleate JN strain likely arose by somatic hybridization of two binucleate isolates, and maintained a diploid nucleus. Homeolog gene pairs in the JN genome have experienced both decelerated or accelerated evolution. Homeolog expression dominance occurred between JN subgenomes, in which differentially expressed genes show potentially less evolutionary constraint than the genes without. Analysis of mating-type genes suggested that *Rhizoctonia* maintains the ancestral tetrapolarity of the Basidiomycota. Long terminal repeat-retrotransposons displayed a reciprocal correlation with the chromosomal GC content in the three chromosome-scale genomes. The more aggressive multinucleate XN strain had more genes encoding enzymes for host cell wall decomposition. These findings demonstrate some evolutionary changes of a recently derived hybrid and in multiple nuclear types of *Rhizoctonia*.

## Abstract (web summary)

Li et al. generate chromosome-scale genome assemblies of the three nuclear types of the basidiomycetous fungi *Rhizoctonia* isolates. Genomic comparisons implicate that uninucleate genomes were hybrids derived potentially from binucleate ancestors, suggesting evolutionary changes of a recently derived hybrid and in multiple nuclear types of *Rhizoctonia*.

## Introduction

*Rhizoctonia* was created as an anamorphic genus in the Basidiomycota. The best-known species, *Rhizoctonia solani* (also known as *Thanatephorus cucumeris*), causes severe damage to more than 200 plant species^1^, while genetic breeding strategies have been limited because only minor- to moderate-effect quantitative trait loci related to resistance to this pathogen have been found thus far^2,3^. The *Rhizoctonia* species reported are mostly multinucleate, but there have been increasing numbers of binucleate and uninucleate *Rhizoctonia* isolates discovered. Both binucleate and multinucleate isolates have been reported to cause stem canker and black scurf on potato plants^4,5^. Uninucleate isolates incite root dieback in forest nurseries on Norway spruce and Scots pine^6^ and have also been isolated from brown patch disease samples of *Festuca arundinacea*^7^. However, *Rhizoctonia* spp. have also been found as endomycorrhizae from medicinal plants of Orchidaceae^8^. *Rhizoctonia* spp. are generally soil-borne phytopathogens with a necrotrophic lifestyle, and the diseases have elevated incidence and severity with increasing adoption of direct-seeding, no-till, and straw-turnover farming techniques^9^.

Somatic hybridization or anastomosis is a process commonly found in filamentous fungi^10^. The hyphal fusion between two different fungal mycelia results in the formation of heterokaryotic cells. Generally, this hyphal fusion triggers the vegetative incompatibility response (VIR) that leads to the death of the fused cell. However, the fusion cells occasionally overcome the VIR to undergo nuclear fusion to generate a single diploid nucleus, which can then form haploid or some aneuploid nuclei followed by mitotic recombination and chromosome loss^10^.

Accumulating information indicate that somatic hybridization of fungal pathogens has an important impact on genetic diversity and adaptation to new hosts. *Verticillium longisporum*, causing Verticillium stem stripe on *Brassica* species, contains a genome composed of two lineages and has conidia that are twice as long but with a narrower host range in comparison with its close relative, *V. dahliae*^11^. *Zymoseptoria pseudotritici* is a recently emerged hybrid species causing disease on wild grasses in northern Iran^12^. The oomycete pathogen *Bremia lactucae*, inciting downy mildew on lettuce, maintains a high incidence of heterokaryotic cells^13^. Genomic analyses of wheat stem rust (*Puccinia graminis* f. sp. *tritici*, Ug99) and wheat leaf rust (*P. triticina*, Pt64) demonstrated that the virulent isolates arose from somatic hybridization and had nuclear exchange between dikaryons^14,15^. The results suggest that hybrid genomes of phytopathogens can provide evolutionary flexibility for the pathogen enabling rapid adaptation to different hosts and other environmental changes.

Classification of *Rhizoctonia* spp. and closely related binucleate species is based on a compatibility system of hyphal fusion into different anastomosis groups (AGs). Binucleate *Rhizoctonia* are grouped into 21 AGs (AG A-U), and multinucleate *R. solani* into 13 AGs (AG1–13)^16,17^. However, the physiology, genetics, and genomics of these predominantly asexually reproducing pathogens are largely unknown. To date, there are draft genomes reports on five multinucleate *Rhizoctonia* isolates belonging to four AGs including AG1-IA and AG8 causing blight on cereal crops etc.^3,18–20^. Variations among the genomes were remarkable from the assembly size to the predicted gene numbers, for instance, 10,489 genes predicted in GD118 (36.94 Mb)^3^, 13,952 genes in WAC10335 (39.82 Mb)^19^, and 11,897 genes in BBA69670 (56.03 Mb)^20^. In previous genome sequencing and assembly efforts using short-reads data^19,20^, the occurrence of multinucleate *R. solani* of primarily heterokaryotic and diploid features were hindrances to complete genome assembly. Therefore, we sequenced genomes of field isolates of *Rhizoctonia* of different nuclear types: uninucleate (JN, SM, and YR)^21,22^, binucleate (LY and RW), and multinucleate (XN), among those JN, LY, and XN were assembled to chromosome-scale. Unexpectedly, each uninucleate isolate had a genome assembly that was twice the size of the binucleate isolate, LY. Genomic comparisons implied that uninucleate genomes (e.g., JN) were hybrids derived potentially from binucleate ancestors.

## Results

### Genome sequencing, assembly, and annotation

Uninucleate (JN, SM, and YR), binucleate (LY and RW), and multinucleate (XN) *Rhizoctonia* spp. were sequenced and assembled with 114- to 265-fold coverage for the six genomes using Illumina reads (Supplementary Tables 1, 2 and Supplementary Fig. 1). To generate more complete assemblies, about 11 GB of the long-reads (average length: ~17.5 kb for JN and LY, 11.5 kb for XN) from PacBio sequencing for each isolate were combined with the short Illumina reads to assemble JN, LY, and XN genomes separately (Table 1 and Supplementary Table 3). Contigs from PacBio reads were obtained independently using HGAP4^23^, Canu v1.5^24^, and MECAT v1.3^25^. Scaffolds were constructed based on contig comparisons using MUMmer v3.23^26^ and corrected using Pilon v1.22^27^ with Illumina paired-end reads. In the final assembly, we obtained 36, 19, and 21 scaffolds for JN, LY, and XN, respectively (Table 1). The assembly size of each genome was at least 98% of the content estimated by *k*-mer analysis, and the total length of the chromosomes accounted for 99.0% or more of each genome assembly (Supplementary Table 4 and Supplementary Fig. 2). Among those scaffolds, 32 JN, 16 LY, and 16 XN scaffolds had putative telomeric sequence repeats 5′-(TTAGG)n-3′ at both F and R ends (Supplementary Tables 5–7), suggesting chromosome-level scaffolding. An orthologous set of 45 transfer RNAs (tRNAs) was found in each of the three genomes (Supplementary Table 8).

**Table 1:** Description of *Rhizoctonia* spp. genome assembly.

Assembly feature	JN	JNa^*^	JNb^*^	LY	XN
Estimated genome size (bp)	97,392,847	/	/	49,083,502	41,800,569
Scaffold count^#^	36	16	16	19	21
Assembly scaffold size (bp)	96,407,277	49,565,071	46,725,450	48,655,841	41,104,060
Scaffold N50 (bp)	3,505,788	3,231,384	3,505,788	3,192,901	2,303,118
GC%	49.47	49.39	49.58	49.43	47.60
Protein-coding genes	29,028	14,978	14,043	14,549	12,349
Chromosomes	32	16	16	16	16
Genes in chromosomes	29,021	14,978	14,043	14,516	12,305
Repetitive regions (%)	19.9	22.81	16.86	20.88	20.82
Heterozygosity (‰)	0.19	0.22	0.16	0.64	2.56

^*^Subgenomes of JNa and JNb were divided from JN genome.

^#^Four scaffolds in JN (total 116,756 bp, predicted 7 genes, 0.12% of assembly size), three scaffolds in LY (253,844 bp, 33 genes, 0.52%), and five scaffolds in XN (400,287 bp, 44 genes, 0.97%) were not assembled into the chromosomes of each genome. Four scaffolds of JN could not be assigned to the JNa and JNb subgenomes.

The number of proteins annotated in XN (12,349) and LY (14,549) were comparable to *R. solani* WAC10335 of AG8 (13,964) and 7/3/14 of AG1-IB (12,268) (Table 1 and Supplementary Tables 9–10). The number of proteins predicted in uninucleate JN (29,028) was nearly double that in binucleate LY, but coincidently all of the three chromosome-level genome assemblies had a gene density of ~3.3 kb per gene. The average predicted gene size in XN was larger than that in LY and JN, due to more and larger introns in XN (Supplementary Table 10). The 6.95 exons per XN gene was more than other *R. solani* isolates sequenced^20^. Protein functions were predicted using the eukaryotic orthologous group (KOG) database. In general, the distributions of different categories of genes were similar in the three strains, with about 45% of genes predicted as “poorly characterized” (Supplementary Table 11).

### Whole-genome duplication of uninucleate *Rhizoctonia* genomes

**Fig. 1:**
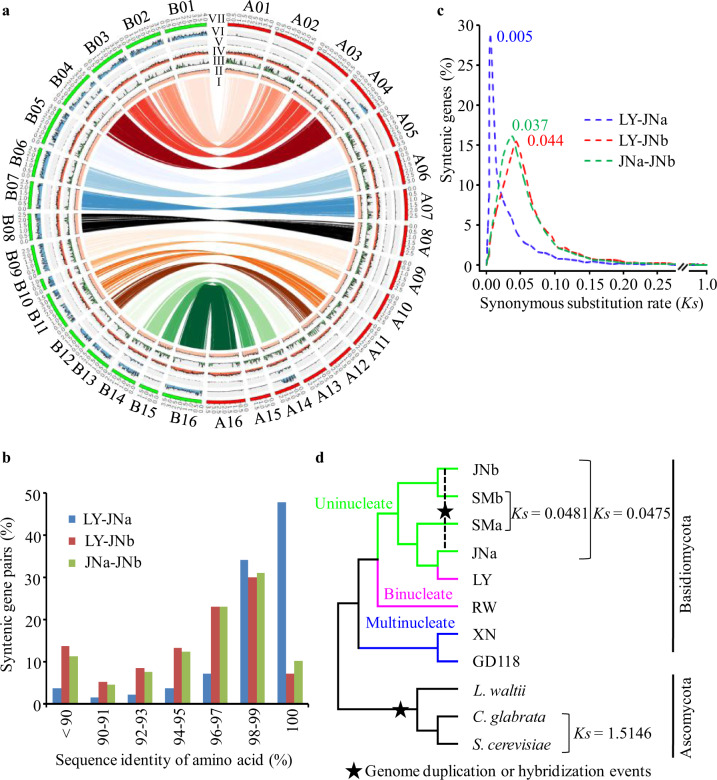
Genomic characterizations and evolutionary relationships of *Rhizoctonia* species. **a** Circos-plot characterization of genomic features of JN. I, for the syntenic gene links; II–VI, representing the frequency distributions of reading coverage (bar 0–150×) repeat density (bar 0–100%), gene density (bar 0–20), hSNP (heterozygous rate, bar 0–10‰), and SNP rate (bar 0–100‰); VII, representing the chromosome karyotypes with red for JNa subgenome and green for JNb subgenome, and the chromosomes numbered with initial of A and B for JNa and JNb, respectively. All statistics are based on non-overlapping windows (window size = 25 kb). **b** Distribution of amino acid sequence identity between the synteny gene pairs among *Rhizoctonia* LY genome and JN subgenomes (JNa and JNb). **c** Distribution of synonymous substitution *Ks* values derived from 8719 one-to-one syntenic gene sets among LY genome and JNa and JNb subgenomes. The peak value for each comparison was shown nearby. **d** Phylogenetic tree was constructed using RAxML (display only topology). Pairwise (*Ks*) rate was estimated based on 637 gene ortholog groups that presented as single copy in the selected genomes or each hybrid subgenome. Selected Basidiomycota *Rhizoctonia*: hybrid uninucleate JN (JNa and JNb subgenomes) and SM (SMa and SMb subgenomes), binucleate LY and YR, and multinucleate XN and GD118 PRJNA51401, and the references of Ascomycota: *Candida glabrata* PRJNA374542, *Lachancea waltii* PRJNA10734, *Saccharomyces cerevisiae*, PRJNA183131. The ratios of *Ks* values between *Rhizoctonia* and the reference species imply the time differences between the genome duplication event in the *Saccharomycetaceae* linage and the hybridization events in *Rhizoctonia*, suggesting about 31 times more recently occurred in *Rhizoctonia*. Extreme values of *Ka* or *Ks* < 0.001 and *Ks* > 10 were excluded from substitution rate analyses.

In comparison with binucleate and multinucleate strains, the uninucleate JN isolate had a larger genome. It was first examined for the intragenomic collinearity, which may be generated by whole-genome duplication (WGD). There were 9,459 (65.2% of the total) gene pairs with synteny in JN (Fig. 1a) versus 496 genes in LY and 464 in XN genome (Supplementary Figs. 3 and 4). Furthermore, 7,919 of the two-copy genes in JN were remarkably larger than that of 151 ortholog groups in the LY genome (Supplementary Table 12). On the other hand, the percentage of single-copy genes was much higher in LY and XN compared with JN (Supplementary Fig. 5). The results imply the occurrence of WGD in the JN genome.

Next, to facilitate genome comparison, we tried to separate the JN genome into two subgenomes. The basic principle was that the protein identity of a JN chromosome as a whole that was closer to binucleate LY was designated as belonging to the JNa subgenome, and those of lower sequence identity were placed with JNb. Therefore, putative subgenome JNa included 16 chromosomes with a total length of 49.57 Mb containing 14,978 predicted coding genes whereas putative subgenome JNb was 46.73 Mb in size, with 14,043 predicted genes (Table 1). Syntenic blocks were determined by MCscan v1.1, setting a minimum stretch containing five genes^28^. The syntenic genes in JN subgenomes against LY genome were 11,685 and 9,058 to JNa and JNb, respectively (Supplementary Fig. 6). As expected, the ratio of identical proteins was higher between LY and JNa in relation to its counterpart JNb (Fig. 1b). The data implied that LY was related to a progenitor of JNa.

Because of the high levels of synteny detected among JNa, JNb, and LY as mentioned above, synonymous substitution (*Ks*) rates were used to infer the occurrence of WGD events that occurred during the evolution of these genomes^29^. The *Ks* distributions showed a paralogous peak at ~0.037 (between JNb and JNa) and an orthologous peak at ~0.044 (between JNb and LY), implying that the divergence time between the JNb progenitor and the LY ancestor was estimated to be around 21 million years (MY). The estimated diploidization of JN was 2.4 MY based on the *Ks* value of 0.005 between JNa and LY (Fig. 1c).

A phylogenetic tree was constructed with *Rhizoctonia* spp. and Ascomycota yeasts as outgroups using 637 single-copy orthologous genes. LY, JN, and SM genomes and subgenomes formed a monophyletic clade with RW, a binucleate *Rhizoctonia* from wheat (Supplementary Fig. 7). The sequence divergence across the hybrid subgenomes was estimated by using the well-characterized WGD in yeasts as a calibration point, as described by Sriswasdi^30^. The sequence divergence across the hybrid subgenomes (*Ks* = 0.0475 for JN and 0.0481 for SM) relative to that of the post-WGD genomes (*Ks* = 1.5146) implied that the *Rhizoctonia* hybridization events were about 31 times more recent (Fig. 1d).

### Differential expansions of LTR-retrotransposons in *Rhizoctonia* spp.

**Fig. 2:**
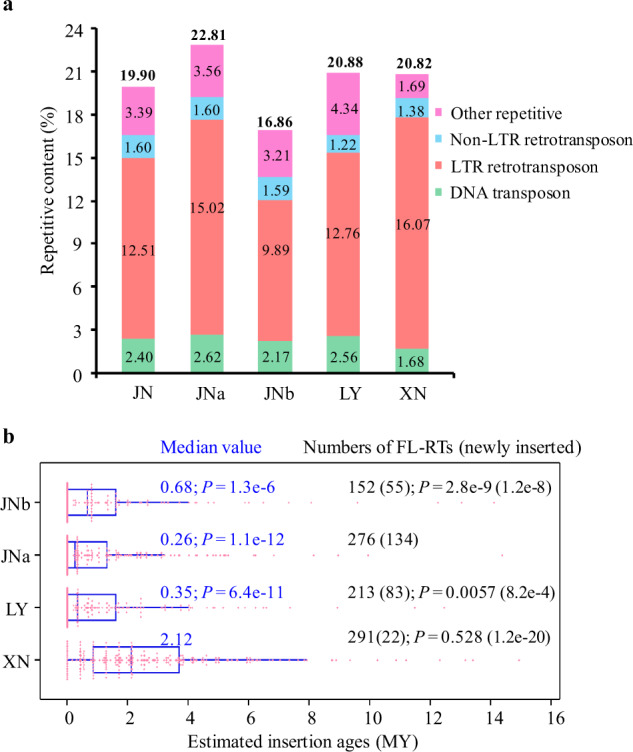
Characterization of repeat elements in the *Rhizoctonia* LY, XN, and JN (JNa and JNb subgenomes). **a** Comparisons of repeat element compositions among genomes of LY, XN, and JN, and divided JNa and JNb subgenomes. Other repetitive representing Low_complexity, Simple_repeat, and Unknown repeat. The numbers in the columns mean the ratio of each repeat element category, and the numbers in bold on the tops of the columns are the total percentages of repeat elements. **b** Distribution of LTR-retrotransposon (LTR-RT) insertion ages in the *Rhizoctonia* data sets. The insertion time of each full-length LTR-RT (FL-RT) was estimated based on the divergence of the 5’-LTR and 3’-LTR ends using the formula “*T* = *K/2r*” (*K* = divergence, *r* = 1.05 × 10^−9^). The median value of insertion ages and *P* values (Student’s *t* test) were shown in the figure with the age range from 0 to 14.93 for XN, 0 to 12.46 for LY, 0 to 14.37 for JNa, and 0 to 14.12 for JNb. The numbers of FL-RTs were also shown with the newly inserted (0 MY) in parentheses and followed by *P* values (binomial test) for comparison calculation against JNa.

The repetitive sequences comprised around one-fifth of each assembly, in which long terminal repeat-retrotransposons (LTR-RTs) were the predominant transposable element (TE) (Fig. 2a and Supplementary Table 13). The estimated TE content was found to be higher in the genome assembly containing PacBio subreads than the Illumina-only assembly (Supplementary Table 14). The content of LTR-RTs in XN was slightly higher than JN or LY, and XN, in which XN contained less TE DNA and uncategorized repetitive elements. There was an uneven distribution of LTR-RTs and full-length LTR-RTs (FL-RTs) in JN subgenomes (Supplementary Tables 14 and 15). The relative ratio of FL-RTs to genomic read abundance of the total LTR-RTs was slightly less in LY, JNa, and JNb compared to XN (Fig. 2b and Supplementary Fig. 8a). The distribution of FL-RTs revealed periodical retrotransposition bursts (Fig. 2b). The median insertion age of FL-RTs in XN was older than that in LY, JNa, and JNb, due to fewer new amplification bursts of LTRs (insertion age 0 MY), indicating more active transposable activity in the uninucleate and binucleate isolates. We found that the number of RNA-Seq reads of LTR-RTs was much more in JNa than XN (Supplementary Fig. 8b). In addition, the number of FL-RTs with 0 MY age also showed a strong relationship with their own RNA-Seq read counts, as well as the RNA-Seq, read counts of total LTR-RTs, suggesting that the greater the number of new LTR-RT insertions, the greater the number of total active LTR-RTs in the genomes reflected in the number of matching RNA-Seq reads. Furthermore, approximately one-third of the JNa intact FL-RTs were syntenic to those of LY (40.4%), and the majority had congruent insertion ages, unlike those of JNb (Supplementary Fig. 9a–c). Phylogenetic analyses of FL-RTs revealed a recent huge expansion branch of LY (103) and JNa (136) in relation to JNb (3) (Supplementary Fig. 10a). In addition, LY and XN showed more independent evolution of FL-RTs (Supplementary Fig. 10b).

### Transition hypermutations in *Rhizoctonia* spp.

**Fig. 3:**
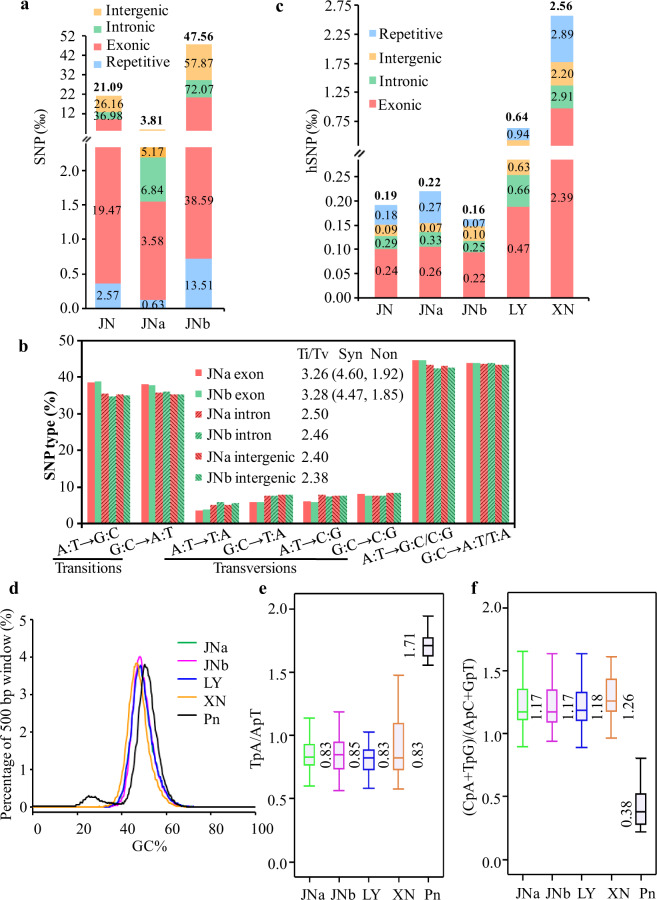
Comparisons of SNP and the heterozygous SNP (hSNP) mutation spectra in *Rhizoctonia* spp. **a** SNP rates of JN or its subgenomes were estimated in relation to LY sequences and divided into four genomic regions with the ratios presented in the figure. The numbers in bold on the tops of the columns are the total SNP ratios. **b** SNP mutation patterns and ratios of transition (Ti) to transversion (Tv) in the data sets. A:T → G:C for A-to-G and T-to-C mutations, A:T → G:C/C:G for A and T-to-G or C mutations, and parity of reasoning for the others. Syn for synonymous variants and Non for nonsynonymous variants. **c** Distribution of hSNP rates in *Rhizoctonia* spp.. The hSNP rates were divided into four genomic regions as described in **a**, and obtained by using SOAPaligner and SOAPsnp software. **d** Distribution of GC contents calculated from 500 bp non-overlapping windows. **e**, **f** The RIP indices of TpA/ApT and (CpA + TpG)/(ApC + GpC) from repeat family of each *Rhizoctonia* data set, respectively. The positive control of *Parastagonospora nodorum* (Pn) showed a bimodal GC distribution and had a strong RIP^7^.

Single-nucleotide polymorphism (SNP) and InDel mutations were estimated between LY and JN. The ratio of SNPs in the JNb subgenome was about 12.5-fold higher than that in JNa, and with slightly lower fold changes in the exonic regions (Fig. 3a and Supplementary Table 16). The pattern of SNP mutations was that the ratio of transition (Ti) to transversion (Tv) was higher in the exonic regions than intronic and intergenic regions (Fig. 3b). The Ti/Tv ratios of synonymous and nonsynonymous variants were very close to those of a set of selected genes observed by Freudenberg-Hua et al.^31^, implying that selection was a major contributor to the Ti to Tv substitution bias.

The process of repeat-induced point (RIP) is one form of genome defense against TE expansion that is active during meiosis^32^. In *R. solani* WAC10335, the heterozygous SNP (hSNP) mutation is considered a RIP-like phenomenon^19^. The hSNP density in XN was the highest, 2.56‰ (i.e., 2.56 SNPs per kb) compared to 0.64‰ in LY, 0.22‰ in JNa, and 0.16‰ in JNb (Fig. 3c and Supplementary Table 17). Mutation patterns indicative of hSNPs were predominantly Ti conversions found in exonic, repetitive or non-repetitive regions (Supplementary Fig. 11a–c). Since RIP-affected genomic regions were often associated with elevated AT content concomitant with a decrease in GC abundance^33^, the distribution of GC content was analyzed and found to be unimodal in the *Rhizoctonia* data sets (Fig. 3d). Furthermore, the RIP indices from the repeat element families of each *Rhizoctonia* genome did not meet the thresholds (Fig. 3e, f), whereas the positive control *Parastagonospora nodorum* met the conditions. The results implied that RIP was not prevalent in the *Rhizoctonia* genomes tested.

### Gene loss, disruption, replacement, and exchange in JN subgenomes

**Fig. 4:**
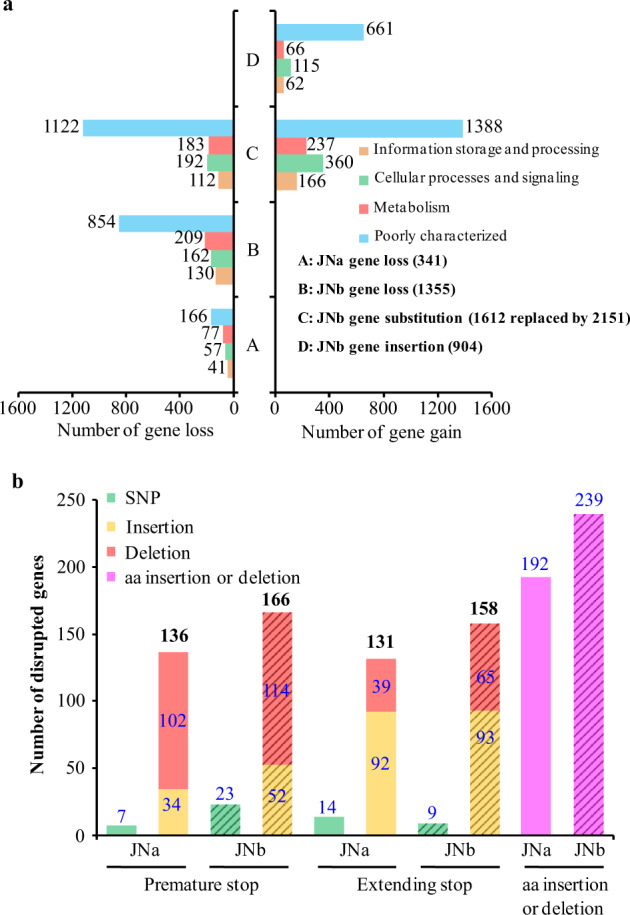
Comparison of gene loss and disruption in the JN subgenomes (JNa and JNb). **a** Gene loss or gain was estimated based on the gene synteny of JNa or JNb in relation to LY. Gene substitution in JNb was that a syntenic fragment was replaced by another fragment containing more or fewer genes in relation to LY. Numbers of gene variations were divided into KOG categories and shown in the figure. **b** Distribution of disrupted genes by SNPs and InDels in JN subgenomes. Gene disruptions were produced by SNPs and nucleotide insertions or deletions, leading to premature or extending stop codons, and amino acid (aa) insertion or deletion compared with the LY syntenic genes. The numbers on the tops of the columns were the total genes modified.

Gene loss, insertion, and replacement in JNa and JNb were assessed in relation to synteny with LY. Gene losses were observed only in JNa (Fig. 4a and Supplementary Table 18), supporting the greater similarity between JNa and LY. However, we observed four-fold levels of gene losses in JNb over JNa, and also large numbers of gene insertions (904) and replacements in JNb (i.e., the gene difference in noncollinear regions of JNa and JNb). The gene-gain phenomenon was estimated to be early events that occurred in the JNb progenitor before the genome hybridization since about half of the genes in JNb had no close homologs in LY or JNa. The largest insertion occurred in chr. B11 of which genomes comprised of 742 kb and the largest deletion was found in chr. B10 affecting about 1.7 Mb (Supplementary Fig. 12a, b). A phenomenon of gene loss took place at one or both chromosome ends of JNb except chr. A15 (Supplementary Table 19). In most cases, TEs were close by the breakage sites, suggesting the association of TEs with the gene loss.

There was a noticeable number of disrupted genes in JN including pseudogenized ones by SNPs and InDels (Fig. 4b). The rate of nonsynonymous to synonymous substitutions (*Ka/Ks*) in the SNPs of exonic regions was relatively higher in JNa than JNb estimated from the whole subgenome or the homeologous regions (Supplementary Table 16), suggesting stronger selection pressure on JNa than JNb. The InDel density in JNb was ~4.2-fold higher than JNa (Supplementary Table 16), however, the gene numbers disrupted by InDels were only slightly more in JNb than JNa, implying higher ratio of gene disruption in JNa (at least in the coding region) (Fig. 4b).

Gene exchanges might have taken place between JN subgenomes and possibly resulted in differences of SNP density in the syntenic fragments. We analyzed the SNP densities of the total 187 homeologous blocks that exhibited uneven distribution in each subgenome (Supplementary Fig. 13). In the case of gene exchanges, the SNP density of b4 in chr. B04 of JNb was lower than its syntenic a4 in chr. A04 of JNa, and exhibited modifications of *Ks* value and amino acid identity in the regions correspondingly (Supplementary Fig. 14a). The data indicated that gene exchanges occurred between the hybrid subgenomes, which were not caused by possible assembly errors since the linkages of the syntenic blocks had normal read coverage. Among the entire subgenomes, there were 245 gene exchange events between the syntenic regions of JNa and JNb, in which 46 exchanged fragments containing multiple genes (Supplementary Table 20). Both chrs. A14 and B14 of the JN subgenomes diverged rapidly from LY with high *Ks* values and low identity of amino acid sequences, whereas both chrs. A15 and B15 showed a high and close identity of protein sequence with LY (Supplementary Fig. 14b, c).

### Differential evolutionary rates and expression dominance among homeolog gene pairs

**Fig. 5:**
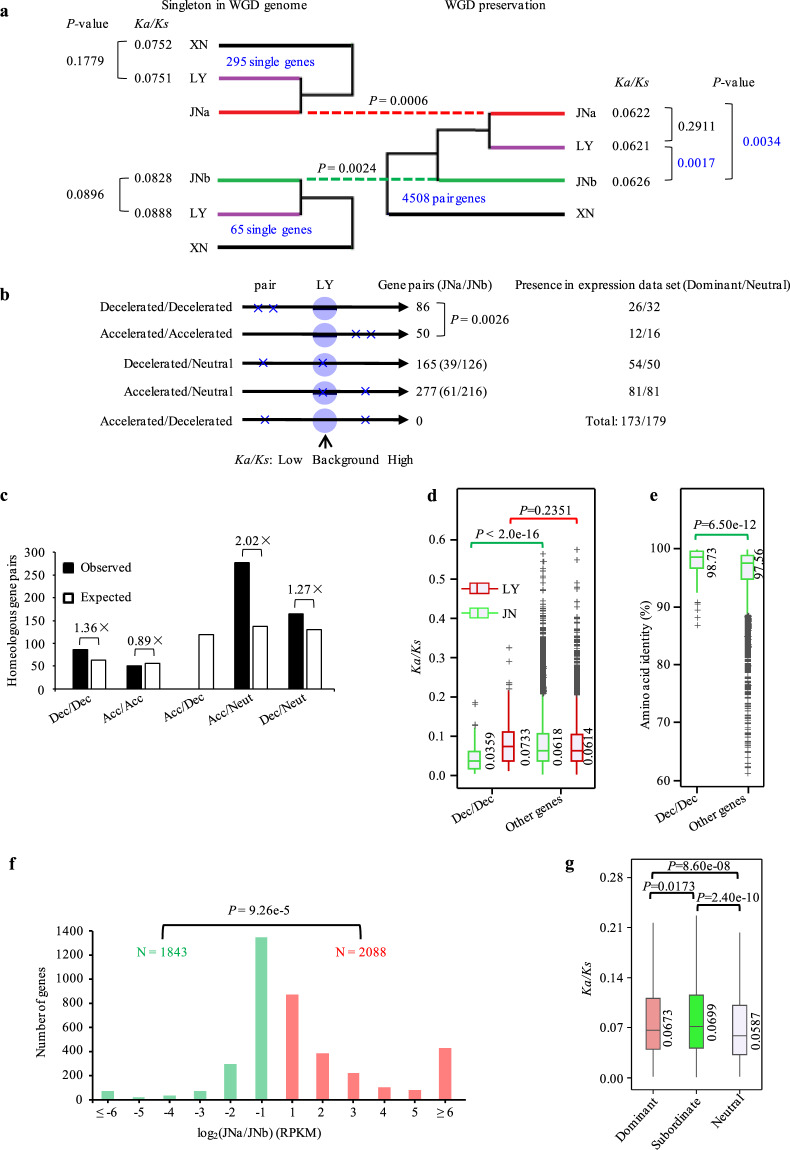
Divergence of evolutionary rates and homeolog expression in JN subgenomes (JNa and JNb). **a** Diagram illustrating gene evolution after whole-genome duplication (WGD) in JN. The gene loss or preservation was analyzed using the multinucleate XN as the ortholog reference and the binucleate LY as the background. The medians of nonsynonymous to synonymous substitutions (*Ka/Ks*) were illustrated in the figure. Significance was evaluated using the Student’s *t* test. **b** Diverged evolutionary rates of the preservation genes in the hybrid JN. The evolutionary rates were compared to the LY background with *Ka/Ks* of ±1.5-fold as the threshold for accelerated or decelerated evolution. The numbers of homeologous gene pairs with evolutionary patterns were shown nearby the arrowheads with JNa/JNb in parenthesis for the gene pairs in each subgenome. The right panel was the gene pairs presenting in the expression data set analyzed in **f**. *P* values according to the binomial test. **c** Comparison of homeologous gene pairs calculated from **b** with those of the expected gene pairs, estimated by the global frequency of each evolutionary pattern. Numbers followed by multiplication signs were the fold variations of gene pairs between observed and expected. Gene pair enrichments with different extents were observed in accelerated/neutral (Acc/Neut), decelerated/neutral (Dec/Neut), and decelerated/decelerated (Dec/Dec) evolutionary patterns. **d**, **e** Boxplots of the distribution of *Ka/Ks* (**d**) and amino acid identity (**e**) between Dec/Dec and the rest of gene pairs (other genes). The *Ka/Ks* values of Dec/Dec homeologs were compared with those of the rest homeologous gene pairs, and the corresponding LY orthologs were used as the references. The cross line in each box represented the median showed nearby. **f** Expression histograms of homeologous gene pairs in JN subgenomes (JNa and JNb). There were total of 7,874 gene pairs, in which 3,931 pairs showed homeologous expression dominance (dominant, 2,088 in JNa and 1,843 in JNb) and 3,943 pairs showed no significant expression differences (neutral). Dominant, subordinate, and neutral refer as higher, lower, and equal expression level in homeolog gene pairs, respectively. *N* for numbers of dominant genes in JNa and JNb. *P* values according to the binomial test. **g** Boxplot showing the distribution of *Ka/Ks* values among homeolog expression dominance genes as dominant, subordinate, and neutral in JN. *Ka/Ks* values were estimated in relation to XN orthologs. There were 2,927 gene pairs that remained in JN for the *Ka/Ks* calculation with 1,137 dominants (623 in JNa and 514 in JNb) and 1,790 neutrals. The median showed nearby the cross line in each box. Significant difference analyses were conducted using the “Mann–Whitney *U* test”.

Evolutionary rates of the preserved duplication genes in JN were calculated with orthologs from the nonhybrid XN genome. The median *Ka/Ks* value of the preservation genes in JNb was higher than LY orthologs and JNa paralogs, whereas the *Ka/Ks* values of the singleton genes in the hybrid genome were not significantly different from their counterparts in LY (Fig. 5a). Evolutionary rates of homeologous genes were analyzed by using the method described by Sriswasdi^30^. The fold differences between the *Ka/Ks* of genes in JN subgenomes and the corresponding background *Ka/Ks* in LY were calculated and categorized using 1.5-fold as a threshold, due to low numbers of the changed genes at a twofold threshold, which showed the same evolutionary patterns (Fig. 5b and Supplementary Fig. 15a). The number of decelerated/decelerated (Dec/Dec) homeolog gene pairs, with decreased *Ka/Ks* values, was more than accelerated/accelerated (Acc/Acc) pairs (Fig. 5b, c). Enrichment of evolutionarily accelerated single homeolog copy (i.e., accelerated/neutral pairs) was consistent with the general evolutionary scenario in which one of the homeolog gene pairs was under less evolutionary constraint. Intriguingly, we did not observe gene pairs with divergent changes in evolutionary rates (accelerated/decelerated pairs).

Analyses of evolutionary rates revealed that both Dec/Dec and Acc/Acc homeologs evolved, respectively, slower and faster than the remaining homeolog pairs in the hybrid genome (Fig. 5d and Supplementary Fig. 15b). Approximately a quarter of Dec/Dec homeologs (21/86) were in the KOG category of information storage and processing, including helicases, transcription and translation initiation factors, and DNA repair proteins (Supplementary Table 21), implying that conserved proteins were prone to decelerated evolution. The protein identity of Dec/Dec homeolog pairs was markedly higher than the other genes (Fig. 5e), but a relevant change was not observed for Acc/Acc gene pairs (Supplementary Fig. 15c), suggesting that gene conversion may have a role in genome stability of young hybrids by creating evolutionary constraints^30^.

To explore the transcriptional behavior of the hybrid subgenomes, we compared the genome-wide transcriptional levels of homeolog genes and found about half of the gene pairs displayed homeolog expression dominance (Fig. 5f and Supplementary Fig. 16a). The homeolog expression dominant genes were classified as dominant, subordinate, and neutral (i.e., higher, lower, and equal expression level in homeolog gene pair, respectively) as previously described^34^. The median *Ka/Ks* and *Ka* values of dominant and subordinate genes were markedly higher than those of neutral genes (Fig. 5g and Supplementary Fig. 16b). The results revealed that the differentially expressed dominance genes evolved faster than the neutral genes, with subordinate genes even faster.

To examine whether sequence evolution had any relationship with the transcriptional evolution, we checked the distribution of the gene pairs (61%, 352) remaining in the expression data set and found no biased proportion of gene losses (Fig. 5b). The homeolog gene pairs in JN showing divergent evolutionary rates in relation to LY were significantly more common (chi-square test, *P* = 1.93e-4) in the dominance expression category (173 in 1,137 gene pairs) than in the neutral expression category (179 in 1,790) (Fig. 5b). The results suggested that homeolog gene pairs of evolutionarily diverged rates were also more divergent in expression levels.

### Identification of mating-type genes in *Rhizoctonia* isolates

**Fig. 6:**
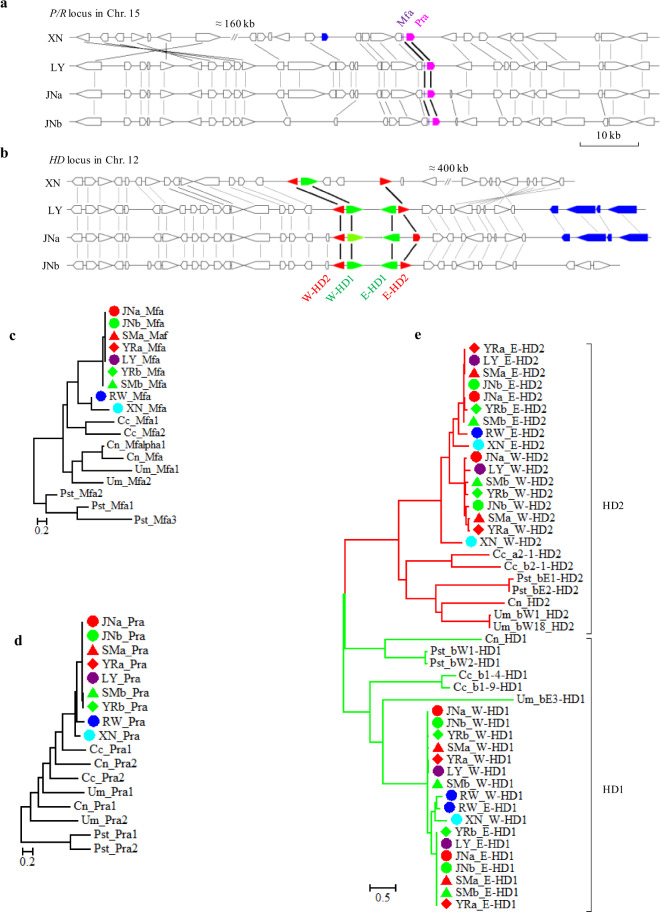
Organization of mating-type loci of *Rhizoctonia* spp. and phylogenetic relationship with other basidiomycetes. **a** Structure and synteny of the pheromone and pheromone receptor (*P/R*) locus in JNa, JNb, LY, and XN. *P/R* locus was located in the chromosome (Chr.) 15 in LY, XN, or JN strain (Chr. 15A for JNa and Chr. 15B for JNb). *Mfa* (purple) and *Pra* (amaranth) for potential pheromone and pheromone receptor genes, respectively; transposase genes in blue; double slash for insertion with the size showed nearby. **b** Collinearity relationship of homeodomain (*HD*) loci in *Rhizoctonia* spp.. *HD* locus was a duplicate in proximity located in Chr. 12, assigned as *W*- and *E*-*HDs* with *HD1* in green and *HD2* in red. **c**–**e** Phylogenetic relationships of *Rhizoctonia* Mfa (**c**), Pra (**d**), and HDs (**e**) with some selected basidiomycete fungi. Pheromone (Mfa) proteins were collected and shown as species_protein name (accession No.): JNa_Mfa (chr. 15A: 993630-749), JNb_Mfa (chr. 15B: 929651-770), SMa_Mfa (scaffold034: 404386-505), SMb_Mfa (scaffold013: 1096589-708), YRa_Mfa (scaffold033: 115988-6107), YRb_Mfa (scaffold085: 70200-319), LY_Mfa (chr.15: 956133-252), RW_Mfa (scaffold0051: 694268-390), XN_Mfa (chr15: 699219-350), *Coprinopsis cinerea* (Cc_Mfa1, XP_002910173; Cc_Mfa2, XP_002910430), *Cryptococcus neoformans* (Cn_Mfa, AAN75621; Cn_Mfalpha1, AAG25675), *Puccinia striiformis*^106^ (Pst_Mfa1, Pst_Mfa2, and Pst_Mfa3), and *Ustilago maydis* (Um_Mfa1, AAA99765; Um_Mfa2, AAA99771). For Pra/SET3-like and HD proteins seeing Supplementary Fig. 17. Phylogenetic trees of mating-type proteins were constructed using RAxML.

Basidiomycete fungi have evolved a mating (MAT) system based on two genetic loci, e.g., the pheromones and pheromone receptors (*P/R*) and the homeodomain-containing transcription factors (*HD*)^15,35,36^. While the P/R system is for haploids to recognize a compatible mating partner, heterodimer formation between HD1 and HD2 originating from different mating types is responsive for postmating events such as the formation of dikaryotic mycelium. Information on mating-type genes in *Rhizoctonia* species is lacking, so we analyzed *MAT* loci across the genomes. The orthologs of *P/R* organization were present for *Rhizoctonia Pra* (*RhiPra*) and the adjacent *RhiMfa* genes (Fig. 6a), which encode for a pheromone receptor and a putative pheromone precursor, respectively. *RhiHD1* and *RhiHD2* genes existed as a duplicate set on the same chromosome, but different from that harboring *P/R* genes (Fig. 6b). Syntenic analyses around *MAT* loci revealed that both *P/R* and *HD* loci were syntenic orthologs between LY and JNa, but arrangements were observed at the one side of JNb_*Pra* and JNb_*E-HD*. Likely, XN had maintained highly co-linearity on one side of the XN*_Pra* or XN_*HD* locus, and the other side with a large fragment insertion and sequence inversion (Fig. 6a, b).

RhiMfa proteins were almost the same in binucleate LY and uninucleate strains with only one amino acid difference in SMb_Mfa and YRb_Mfa, but highly divergent with binucleate RW and multinucleate XN (Fig. 6c and Supplementary Fig. 17a). Similar divergences were detected among the RhiPra pheromone receptors, showing marked variations among LY, RW, and XN (Fig. 6d). In the *RhiHD* locus (Fig. 6e), the copies of W-HD1 and W-HD2 were apparently intact except for lack of RW_W-HD2, whereas E-HD1 and E-HD2 varied in the loss of XN_E-HD1, and an N-terminal shortening of LY_E-HD1 and JNa_E-HD2. The *Rhizoctonia P/R* and *HD* genes were phylogenetically closer to corresponding genes of the ink cap mushroom *Coprinopsis cinerea* than to plant pathogenic genera, *Ustilago* and *Puccinia*, in agreement with the phylogenetic tree of these species generated using 889 single-copy genes (Supplementary Fig. 18). Allelic-like variants of *RhiPra* were present in each (sub)genome; however, their adjacent *RhiMfa*-like genes were absent. In addition, the *RhiPra* and *RhiHD* variants were in (sub)clades different from the potential *MAT* genes (Supplementary Fig. 17b, c), implying divergent evolution of these genes. Taken together, *Rhizoctonia* spp. maintain the ancestral tetrapolarity of Basidiomycota.

### The divergence of XN vs. LY or JN

Although XN and LY genomes remained high levels of synteny for orthologous genes, dynamic rearrangements of the genes were observed when comparing XN and LY genomes (Supplementary Fig. 19). For instance, the rDNA operons were assembled in two copies in chr. 14 of LY and JN (sub)genomes, but the rDNA repeats were in chr. 03 of XN (Supplementary Fig. 20). However, the number of rDNA operon repeats should be at least 20 times greater estimated from the read coverage. Also, rearrangements were identified around *MAT* loci as mentioned above, even though the regions were shown to have lower recombination rates in some fungal species^15^.

**Fig. 7:**
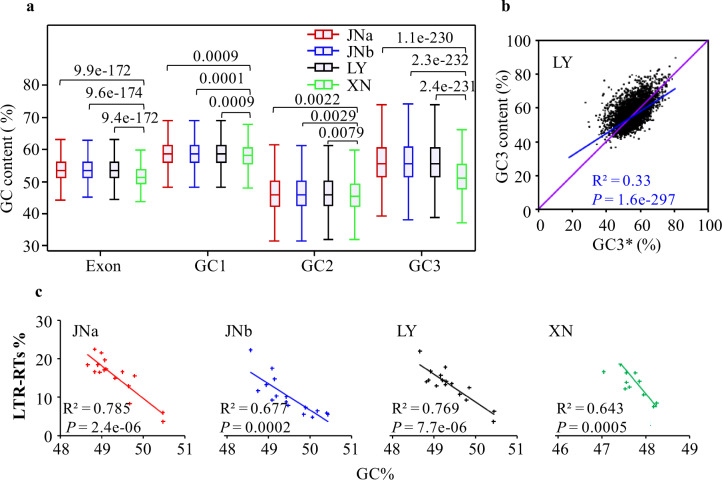
Comparison of GC contents among JNa, JNb, LY, and XN. **a** The exonic GC content of XN orthologous genes (4,508) was markedly different from that of LY, JNa, or JNb, and the difference was predominant from the third codon positions (GC3) in relation to the first (GC1) and second codon positions (GC2). The medians and the ranges were following: exon XN 51.36 (42.26–68.75), JNa 53.39 (44.07–70.38), JNb 53.44 (40.78–70.99), LY 53.38 (44.07–70.54); GC1 XN 57.38 (37.36–72.12), JNa 58.68 (40.16–73.75), JNb 58.67 (32.94–74.39), LY 58.66 (40.16–73.86); GC2 XN 45.52 (27.03–67.34), JNa 45.91 (22.56–69.36), JNb 45.90 (22.56–69.42), LY 45.93 (22.56–69.57); GC3 XN 50.98 (34.18–80.12), JNa 55.50 (36.72–89.57), JNb 55.57 (37.85–88.10), LY 55.50 (36.72–90.05). *P* values were shown above the connected comparisons and estimated according to the Student’s *t* test. **b** Correlation between the frequency of GC3 and equilibrium GC3 content (GC3*), reflecting the AT to GC substitution rates of LY compared with XN orthologs at the third codon positions. GC3* value was calculated as the ratio between the AT to GC substitution rates (XN to LY) using the model of Sueoka^107^. GC3* = u [(AT→GC) + (AT→CG)]/[(AT→GC) + (AT→CG) + (GC→AT) + (GC→TA)]. The diagonal line in purple and the correlation line in blue. *P* values were calculated using Kendall’s rank correlation. **c** Reciprocal relationship was observed between chromosomal GC% and LTR-RTs content in JNa (*y* = −8.397*x* + 429.6), JNb (*y* = −6.884*x* + 350.8), LY (*y* = −7.156*x* + 366.6), and XN (*y* = −13.60*x* + 663.7).

XN, LY, and JN also exhibited genomic differences such as genome size, GC content, and gene numbers (Table 1). GC contents of XN were lower than LY and JN in both exons and intergenic regions (Supplementary Fig. 21), in which exonic GC% was mainly contributed by the third codon (GC3) position (Fig. 7a). Analyses of the codon usage indicated that XN had similar patterns as LY and JN, but with less fluctuation (Supplementary Fig. 22a, b). The preferred codons for the three genomes, for example, CGC (Arg), ATC (Ile), CTC (Leu), TCG (Ser), and GTC (Val), all ended with C or G, and coincidently the codons at the troughs were the same amino acids but with lower GC composition ending with A or T. Intriguingly, there were no corresponding decoding tRNAs for these C-ending codons mentioned above (Supplementary Table 8). The data imply that the adenosine in the first position of the tRNA anticodon may be modified through deamination to inosine, which can wobble with codons ending in A, C, or T^37^. Furthermore, we calculated the equilibrium GC3 content, GC3*, reflecting the AT to GC substitution rates of LY and JN compared with XN orthologs at the third codon positions. GC3 was strongly correlated with GC3* and distributed slightly above the diagonal lines for LY, JNa, and JNb (Fig. 7b and Supplementary Fig. 23), supporting the hypothesis that the *Rhizoctonia* lineages faced similar selection pressures for codon usage. However, LY and JN slightly favored fixation of GC over AT in comparison with XN, consistent with the resulting lower GC content in the XN genome.

The abundance of LTR-RTs was another potential factor influencing genome GC content since the level of LTR-RTs was negatively related to gene density in a chromosome^38^ (Supplementary Fig. 24a). Also, the chromosomal GC contents showed a high reciprocal correlation with the abundance of LTR-RTs or TEs, and a positive relationship with the gene density in the data sets (Fig. 7c and Supplementary Fig. 24b, c). LTR-RT content possibly posed a small contribution to decreased GC% due to the LTR sequences having lowered GC content than that of the genome or of exonic regions (Table 1 and Supplementary Fig. 8).

### Gene expansions for degradation of cell wall components in XN genome

**Fig. 8:**
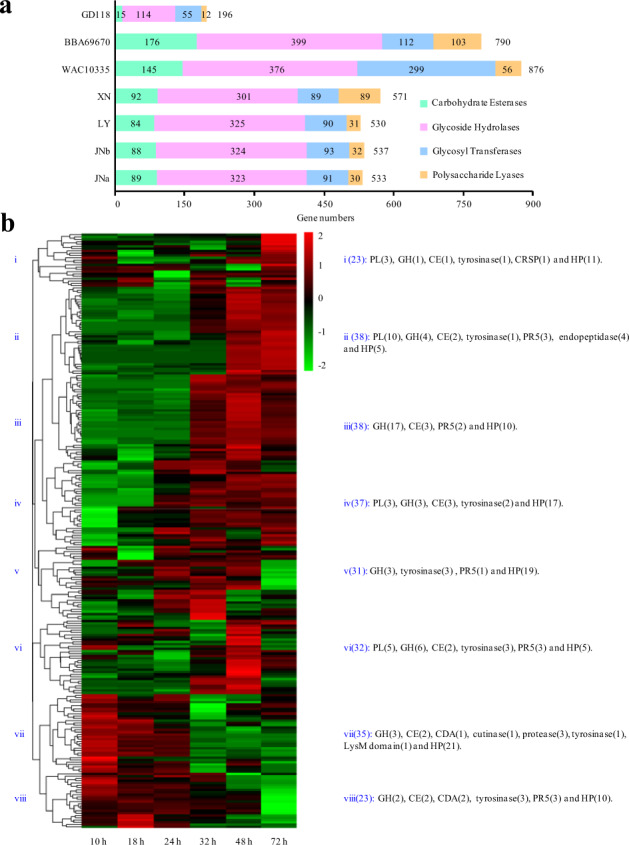
Comparisons of CAZyme genes in *Rhizoctonia* spp. and expression of effector candidate genes in XN. **a** Comparison of carbohydrate-active enzyme (CAZyme) genes was conducted with BBA69670^20^, WAC10335^19^, GD118^9^, and the data sets in this study. **b** Expression patterns of potential effector genes of XN were analyzed using the RNA-Seq data of GD118-infected rice plants at 10, 18, 24, 32, 48, and 72 h post inoculation (BioProject, PRJNA147097). In all, 257 of 354 XN effector candidate genes were mapped by the GD118 RNA reads and used in the hierarchic analyses. The main CAZyme and some selected genes followed by gene numbers in parentheses were listed in each group. CDA chitin deacetylase, CE carbohydrate esterase, CRSP cysteine-rich secretory family protein, GH glycoside hydrolase, HP hypothetical protein, PL polysaccharide lyase, PR5 pathogenesis-related protein PR5.

Multinucleate *Rhizoctonia* isolate was more aggressive than binucleate or uninucleate isolate^22^. To examine characteristics of *Rhizoctonia* spp. thriving on dead or dying plant cells and explore why the XN strain was more aggressive than LY or JN, CAZyme (carbohydrate-active enzyme) genes were investigated to see whether a particular set of enzymes was associated with host range and pathogenesis. As shown in Fig. 8a and Supplementary Fig. 25, the XN genome had experienced an expansion and diversification of polysaccharide lyases (PLs), which mainly degrade pectin and glycosaminoglycans. Compared with other *Rhizoctonia* genomes, XN had many fewer CAZyme members than BBA69670 of AG2-2IIIB^20^ or WAC10335 of AG8^19^, but many more than GD118, which is of the same AG1-IA as XN^3^.

Secreted and effector proteins of plant pathogens are required for establishing successful infection and evading host defense responses during colonization. Secreted proteins in XN were fewer than those in LY and JN (Supplementary Table 22). Effector proteins in the secretome of XN (354) were slightly fewer than in LY (393) or JN (399/384, JNa/JNb). Expansion of effectors in XN (≥five than in LY) in relation to LY were PLs, polygalacturonases, and tyrosinases, on the contrary, LY had more alpha/beta hydrolases and hypothetical proteins (HPs).

Making use of the available RNA-Seq data from GD118-infected rice^3^, we found that most of the genes encoding CAZymes (93%), secreted proteins (87%), and effector candidates (73%) could be mapped by the Illumina reads from GD118, suggesting that both XN and GD118 maintained genes of fundamentally the same characteristics. A closer examination of the effector genes showed that the unexpressed genes in XN (97) related to GD118 reads were mainly HPs (57), PLs (21), tyrosinases (6), or polygalacturonases (5). A possible explanation is the expansion or gain of these genes after the split of the two strains since a high proportion of the unexpressed genes in GD118 had detectable expression in XN RNA-Seq. From the phylogenetic tree, clades of vii, viii, and including i with the genes of early high expression were enriched for HPs, but also contained several proteases of various kinds, tyrosinases, three chitin deacetylases (CDAs), and the only LysM domain protein of effector candidate (Fig. 8b). The LysM domain protein and CDAs may act on early pathogenesis through binding and modification of chitin oligomers to suppress chitin-induced immunity^39,40^. In the subclades of ii and iii with the genes highly expressed in the later infection times, enrichments of the GH family in iii and PLs genes in ii revealed that the encoding enzymes may function on the degradation of the plant cell wall. The results indicate strongly that various effectors and secreted enzymes participate in different processes of *Rhizoctonia* infection host plants.

## Discussion

Hyphal fusion is presumed to be common in *Rhizoctonia* since the anastomosis compatibility system is used to differentiate taxa. However, genomic information on hyphal fusion in *Rhizoctonia* spp. is incomplete. In this study, we identified naturally occurring uninucleate *Rhizoctonia* isolates as hybrid genomes which maintained in their diploid nuclei. Hybrid fungal species have been shown in *Saccharomyces*, *Cryptococcus*, *Verticillium*, and *Puccinia*^11,14,30,41^. The fused fungal hyphae normally restore the haploid state from the hybrid diploid nucleus by chromosome loss^10^. Occasionally, the hybridized nuclei are stable, resulting in a hybrid species with a diploid or aneuploid nucleus. *Epichloë* polyploid Lp1 is a well-characterized asexual interspecific hybrid between sexual and asexual Epichloë species^42^. The endophyte Lp1 maintains most of both parental gene copies with the notable exception of uniparental rDNA repeats^42,43^. Here, the uninucleate JN genome still contained both parental rDNA operons, suggesting recent hybridization or independent evolution of the rDNA loci in the hybrid lineage^43^.

Mating phenomena of *R. solani* have been studied by pairing cultures of mycelia generated from single-basidiospore isolates or deduced from single-protoplast isolates because of difficulty in triggering sexual reproduction in this species complex^44^. The mating system of *R. solani* is unclear because there are reports of homothallism or heterothallism with bipolar mating in several AGs, and both homothallic and heterothallic mating systems in an AG2-2 IV isolate^45^. We found that the *Rhizoctonia P/R* and *HD* loci not only had a similar organization to those of *C. cinerea*, except for the absence of repeats but also the genes were phylogenetic closer than other species tested^35,46^ (Fig. 6). Like in *C. cinerea*, additional non-mating-type-specific receptors and multiple alleles of *HD* genes also exist in the *Rhizoctonia* isolates (Supplementary Fig. 17). This information suggests that *Rhizoctonia* spp. may have a tetrapolar mating system similar to *C. cinerea*. In the hybrid uninucleate genomes, the presence of two sets of almost identical *P/R* and highly homologous *HD1/HD2* genes revealed that the fungal fusions overcame the VIR and persisted into a parasexual cycle. The switch from sexual to asexual only requires a single-nucleotide change within a key gene of *MAT* locus, or some mutation involved in mating or meiosis^47^. It is unclear what maintains the diploid state of uninucleate strains, which may be stable because the hyphae with diploid nuclei continue to grow. Nevertheless, our data provide valuable information to pursue the hyphal fusion and mating phenomena in the predominant asexual *Rhizoctonia* species.

The genome modifications of JN, especially in JNb, were composed of two parts: divergence between the unknown parents before the hybridization and post-genome hybridization events. For instance, a large proportion of the arranged JNb-genes had no homeologs in LY or JNa (Fig. 4a), implying that these genes did not come from JNa or LY but from the unknown parent. The faster evolutionary rate of JNb than LY or JNa (Fig. 5a) may be a source of this variation. Gene loss was expected to be high in the new hybrid genome. However, the number of gene loss events in JNa was markedly less than the number of genes disrupted by SNPs and InDels (Fig. 4b), indicating that sub- and neo-functionalization and pseudogenization of genes played active roles in the modification of the hybrid genome^48^. The evolution of genome architecture is thought to be highly associated with TEs, particularly LTR-RTs, since TEs are the major driving force for genome evolution through their movement and proliferation^38^. Increased copy number and transcriptional abundance of LTR-RTs especially in JNa subgenome are speculated to be genomic shock post the hybridization^49^ (Fig. 2 and Supplementary Fig. 8), which can elevate mutation rates through activated TEs, as documented in hybrid fish and sunflower^50,51^.

On the other hand, TEs impact the gene content of a genome by rearrangements through homologous recombination and by insertion or excision, which causes double-strand DNA breaks (DSBs)^52^. DBSs are repaired by homologous recombination and non-homologous end-joining (NHEJ) mechanisms, in which NHEJ is an error-prone process not only leading to nucleotide fixation but also causing genetic material deletions, inversions, and translocations^52–54^. Bold speculation is that earlier expansion of LTRs in the XN genome is possibly one of the driving forces leading to variations of GC content and gene order between XN and LY. Also, chromosomal rearrangements have been found as a general mechanism for host adaptation of the asexual pathogen *V. dahllium* which established the lineage-specific genomic regions mediating aggressiveness^55^.

In the hybrid JN genome, we observed not only a dominant relaxed selection on one copy of the homeologous gene pairs but also gene enrichment of Dec/Dec evolutionary rates (Fig. 5c–e), suggesting that gene conversion likely played a role in maintaining the functional integrity of redundant genes^30^. Homeolog expression dominance is a consequence of eukaryotic hybrids and varies markedly across different hybrid species^34,56,57^. We observe that the subordinate expression genes in the homeolog expression dominance were evolutionarily faster, in consistent with accelerated genes being mainly in the subordinate group of low expression levels (Fig. 5g). This phenomenon also agrees with that observed in the allopolyploid plant *Brassica juncea*^34^. The evolutionarily diverged homeolog genes tended toward expression dominance (Fig. 5g), suggesting that the sequence diverged homeolog pairs would be divergent in their expression dominance. However, these findings and analyses were preliminary, and future work should focus on comparisons and more in-depth analyses to explore the evolution of *Rhizoctonia* and evaluate species boundaries.

The expanded gene families, such as PLs, polygalacturonase, and tyrosinases in XN possibly played important roles in its pathogenicity, since the tested strains contained similar kinds of secreted proteins and effectors with different virulence. Polygalacturonases, cleaving the linkage of galacturonic acid in pectin, have been characterized as virulence effectors in plants^58–60^. The expanded PLs are mainly pectate lyases PL1 and PL3, predominantly degrading the poly-galacturonan regions of pectin. Tyrosinases, functioning in plant biomass decomposition and also in melanin biosynthesis, showed wide expression patterns probably associated with multifaceted roles. The early *Rhizoctonia* infection could be hemibiotrophic as previously suggested^61,62^, whereas the late process of massive necrosis required more and active CAZymes and other enzymes to decompose host cell wall components. It is plausible that the more aggressive XN compared with LY and JN is due to XN evolving more genes or active enzymes for the late necrosis. Efforts are being made to facilitate molecular manipulation of *Rhizoctonia*, and to characterize effector candidates.

## Methods

### Collection of isolates

*Rhizoctonia* isolates JN, YR, LY, and XN were collected from maize as previously described^21,22,63^, and isolate RW from wheat was kindly provided by Dr. W. Li^64^. Isolate SM was obtained from a blighted maize sheath near Beijing, China. All *Rhizoctonia* isolates were further purified by a single-protoplast procedure and kept on potato dextrose agar slants covered with glycerol at 4 °C for further use. The rDNA-ITS sequences of each purified isolate were compared with those of standard strains for the determination of the anastomosis group (Supplementary Table 1). Nuclear status was assessed by 4′,6-diamidino-2-phenylindole, dihydrochloride (DAPI) staining^21^.

### Illumina and PacBio genomic DNA sequencing

Mycelia of each isolate were cultivated in potato dextrose broth at 28 °C for 72 h with shaking (150 rpm) and collected for genomic DNA isolation using CTAB. For Illumina sequencing of JN, multiple DNA libraries, paired-end (180 and 500 bp) and mate-pair (2, 5, and 10 kb), were used. Genome sequencing of LY, XN, YR, SM, and RW included two mate-pair sequencings (2 and 5 kb) and plus one 500 bp paired-end library for LY and XN or a 350 bp paired-end one for each of the remaining genomes. All DNA libraries were sequenced on an Illumina HiSeq 2000 system by BGI, Shenzhen, China. The raw reads were filtered using Trimmomaticv 0.32^65^ by removing bases with a quality score of 25 or less, adapter sequences, possible contaminated reads, and reads less than 75 bp in length to obtain high-quality reads. For PacBio sequencing, ~20 kb libraries for JN, LY, and XN were prepared at Novogene Bioinformatics Technology Co., Ltd. After sequencing, the subreads were filtered using SMRTlinkv5.0 (-minReadScore = 0.8 and -minLength = 1000). SOAPec_v2.01^66^ was used for genome size estimation with “Genome size = kmer_Number/Peak_Depth”.

### RNA sequencing and analysis

Mycelia of JN, LY, and XN were grown in 500-mL flasks containing 150 mL of potato dextrose broth at 28 °C for 48 h, and collected for RNA isolation. RNA Illumina sequencing was performed at BGI. The cleaned reads, after removing adaptor, empty tag, and low-quality sequences, were aligned to the respective assembled genome using TopHat v2.0.14^67^. Transcript abundance (FPKM, fragments per kilobase of exon per million fragments mapped) was quantified using Cufflinks v2.0.0^68^. The differentially expressed genes were examined using Cuffdiff within the Cufflinks program^68^. Expression patterns of potential effector genes of XN were analyzed using the RNA-Seq data of GD118-infected rice plants at different time points post inoculation (BioProject, PRJNA147097).

### Assembly and annotation of the genomes

The high-quality Illumina cleaned reads were assembled using SOAPdenovo v2.04^66^ (parameters command line: SOAPdenovo-127mer all -s config.txt -F -K 23 -p 50 -o out_put.) and SSPACE v3.0^69^ for scaffold construction, and GapCloser_v1.12^66^ for gap filling. High-quality PacBio subreads of JN, LY, and XN were assembled using HGAP4^23^ (parameters: algorithm options (--minMatch 12 --bestn 10 --minPctSimilarity 90.0), minimum concordance = 70, minimum length = 5 kb, seed coverage = 50, genome length = 95000000 (45000000 for LY and XN)), Canu v1.5^24^ (parameters command line: canu -p canu_out genomeSize = 95 m useGrid = false -pacbio-raw subreads.fasta.gz gnuplotTested = true stopOnReadQuality = false maxThreads = 50, genomeSize = 45 m for LY and XN.), and MECAT v1.3^25^ (Step 1, using mecat2pw (default parameters) of MECAT to detect overlapping candidates. Step 2, correct the noisy reads based on their pairwise overlapping candidates mecat2cns command (default parameters) of MECAT. Step 3, extract the longest 25X corrected reads using extract_sequences (default parameters) of MECAT. Step 4, assemble the longest 25X corrected reads using mecat2cacu of MECAT (mecat2canu ErrorRate = 0.02 maxMemory = 40 maxThreads = 50 useGrid = 0). The assembled PacBio genome contigs were corrected using Pilon v1.22^27^ with Illumina paired-end reads. The telomeric repeats (TTAGGG/CCCTAA) were used to assess integrity at both ends of scaffolds^70^.

### Identification of SNP and heterozygosity

Genome analysis tool kit 1.6 (GATK) tools^71^ and Samtools^72^ were used for SNP and InDel analysis. SNPs and InDels of JN were called using the Illumina reads of LY, the results were filtered as “quality sites (QUAL) < 30, QualByDepth (QD) < 2.0, Fisher strand (FS) > 60.0, RMSMappingQuality (MQ) < 40.0, MQRankSum < −12.5, and ReadPosRankSum < −8.0” for SNPs and “QUAL < 30, QD < 2.0, FS > 200.0, and ReadPosRankSum < −20.0” for InDels. The genome heterozygosity was calculated using SOAPaligner v2.21 and SOAPsnp software v1.03^73^, with the following filters: quality score of consensus genotype ≥20, rank-sum test *P* value >0.05, and minor allele count (supported by ≥ 5 reads).

### Repetitive sequences and RIP analysis

Repeat sequences of the genome assemblies were identified using the RepeatModeler (URLs: http://www.repeatmasker.org/RepeatModeler.html), LTR-FINDER v1.0.6 and LTR_retriever v2.8.2^74,75^. The insertion age of full-length LTR-RT was estimated by using the formula *T* = *K/2r*, where *“T”* is insertion time, *“K”* is the divergence level between the 5′- and 3′-LTRs, *“r”* is the fungal substitution rate (1.05 × 10^−9^ nucleotides per site per year)^76^. Repeat-induced point (RIP) mutations were predicted with the RIPCAL program v1.0.5, setting indices of (TpA/ApT) ≥0.89 and (CpA + TpG)/(ApC + GpT) ≤ 1.03 as the indicative of RIP presence^77^. A necrotrophic pathogen *Parastagonospora nodorum* was used as the positive control of RIP^78^.

### Analyses of mating-type loci

The mating (MAT) type genes of *Rhizoctonia* spp. were obtained by BLAST searches of their genomes against MAT proteins of basidiomycetes from NCBI, including the pheromones (Mfa) and pheromone receptors (STE3 or Pra), and homeodomain (HD) transcription factors. MCscan v1.1^28^ was used for analyses of gene synteny of the *MAT* loci of *Rhizoctonia* isolates.

### Gene prediction and functional annotation

Three de novo gene prediction programs, Augustus v2.7^79^, GeneMark+ES v4.0^80^ and SNAP v2013-02-16^81^, were used to predict the protein-coding regions of *Rhizoctonia* assemblies in combination with homology-based and RNA-Seq sequence mapping. Protein sequences from previously sequenced *R. solani* genomes were also used. These included GD118 of AG1-IA^3^, 7/3/14 of AG1-IB^82^, BBA69670 of AG2-2IIIB^20^, WAC10335 of AG8^19^, and Rhs1AP of AG3^18^, which were mapped to our genome assemblies for homology-based gene prediction using Exonerate v2.2.0^83^ (using options -percent 50 -showtargetgff -m protein2genome -n 1). All RNA-Seq reads were aligned to the genome assemblies with Tophat v2.0.14^67^, and transcript assembly was conducted with Cufflinks v2.0.0^68^. The final gene models were derived through EvidenceModeler v2012-06-25^84^ integration. Functional annotations of the predicted genes were performed by BLAST and HMMER searches against the NCBI GenBank non-redundant, CAZy (carbohydrate-active enzymes), KOG, and Pfam databases. The tRNA genes were detected using tRNAScan-SE v1.3.1^85^.

### Secretome and effectors prediction

Secreted proteins and effector candidates of JN, LY, and XN were analyzed using TMHMM Server v. 2.0^86^ and Phobius 1.01^87^ for prediction of the transmembrane domains, SignalP 4.1 Server^88^ and PrediSi^89^, and TargetP 1.1 Server^90^ for subcellular location and signal peptide cleavage sites. Glycosylphosphatidyl inositol anchor proteins were excluded by PredGPI^91^. For more comprehensive and accurate effector prediction, we took into account the predicted secretory proteins of those less than 400 amino acids and less than four cysteines using the Klosterman standard^92^, and we used the fungal effector predictor program, EffectorP 2.0^93^.

### Whole-genome duplication and subgenome reconstruction

Syntenic blocks in each species and between JN and LY were identified by MCscan v1.1^28^ using parameters of MATCH_SCORE 50, MATCH_SIZE 5, GAP_PENALTY -1, OVERLAP_WINDOW 5, E_VALUE 1e-15, MAX GAPS 5, IDENTITY 50%, and COVERAGE 70% and synteny distributions were plotted using Circos^94^. Synonymous (*Ks*) and nonsynonymous (*Ka*) substitutions values of syntenic genes were calculated using the YN model^95^ by MAFFT v7.221^96^, ParaAT v1.0^97^, and KaKs_Calculator v1.2^98^. The *Ks* distributions were plotted to estimate speciation and whole-genome duplication events^99^. The time was estimated at the peak value by using the “*t* = *Ks/2r*” formula, which was used to estimate the divergence time between two species genome, where “*Ks*” is the peak value of *Ks* distributions, “*r*” is the fungal neutral substitution rate (1.05 × 10^−9^)^76,99^.

Subgenomes of JNa and JNb were divided from JN using LY genomic sequences as the reference, and the same for reconstructions of SM and YR subgenomes. Scaffolds of homeologs were compared to LY, and scaffolds of higher sequence identity with LY were assigned to subgenome a and the rest to subgenome b. Genomic modifications in JN subgenomes were analyzed through the syntenic genes among JNa, JNb, and LY. Given the synteny blocks of JNa, JNb, and LY having the same gene orders, changes of co-linear JNa and LY against JNb represented modifications in JNb, including gene loss, gene gain, and gene replacement, versus changes of co-linear JNb and LY against JNa as JNa variations. Amino acid identity and *Ks* value of syntenic gene pairs were used to find exchanged genes between the two subgenomes JNa and JNb using LY as the reference. Gene exchange between JN subgenomes was defined by comparison of amino acid identity values (*Ks*) among JNa, JNb, and LY, exchanges were considered to have occurred when the *Ks* of a JNb protein was closer to LY than that of JNa.

### Evolution rate calculation

Multiple sequence alignment for each single-copy orthologs gene family of four different (sub)genomes (XN, LY, JNa, and JNb) were carried out using MAFFT v7.221^96^, and the ML phylogenetic tree using RAxML v8.1.24 (randomized accelerated maximum likelihood)^100^. Then, a codon multiple alignments was created from the protein sequence alignment result using ParaAT v1.0^97^ for estimation of evolutionary rates (*Ka*, *Ks*, and *Ka/Ks*).

### Identification of orthologous genes and phylogenetic reconstruction

OrthoMCL v2.0.9^101^ and all-versus-all BLASTP (E-value ≤1e-15, coverage ≥50%) were used to identify orthologous groups for JNa, JNb, SMa, SMb, LY, XN, RW, GD118^3^, *Candida glabrata* DSY562^102^, *Saccharomyces cerevisiae* YJM1078^103^, and *Lachancea waltii* NCYC 2644^104^, in which the Ascomycetous yeasts were used as references for whole-genome duplication^30^. Single-copy orthologs were extracted using Perl script (command line parameters of Gblocks: Gblocks proteins.fasta -b4 = 5 -b5 = h.) and subjected to global alignment using MAFFT v7.221^96^, in which the poorly aligned regions of concatenated sequences were removed by using Gblocks v0.91b^105^. The final phylogenetic tree was constructed using RAxML v8.1.24^100^.

### Statistics and reproducibility

All statistical analyses and visualization were performed using R Project, online OmicShare tools (https://www.omicshare.com/tools), and MATLAB. Statistical significance was done with various tests, including Student’s *t* test, binomial test, chi-square test, and Mann–Whitney *U* test, as well as Kendall’s rank correlation.

### Reporting summary

Further information on research design is available in the Nature Research Reporting Summary linked to this article.

## Supplementary information

Supplementary Information

Reporting Summary

## Data Availability

The genome assemblies described in this paper are the first versions. The whole-genome assemblies and sequence data of *Rhizoctonia* spp. JN, LY, and XN described here are available and have accession numbers assigned in NCBI BioProjects PRJNA624246, PRJNA624247, and PRJNA624219, respectively.
