# Supplementary material for: Evolutionary and genomic comparisons of hybrid uninucleate and nonhybrid Rhizoctonia fungi
Source: Commun Biol. 2021 Feb 15;4:201. doi: 10.1038/s42003-021-01724-y (PMC7884421; doi:10.1038/s42003-021-01724-y)
Supplement: Supplementary file 1 — Supplementary Information [file 42003_2021_1724_MOESM1_ESM.pdf]

Supplementary Information

Evolutionary and genomic comparisons of hybrid uninucleate and nonhybrid *Rhizoctonia* fungi

Cheng Li[1], Zejian Guo[1], Shanyue Zhou[2], Qingyue Han[1], Manman Zhang[1], You-liang Peng[1], Tom Hsiang[3] & Xujun Chen[1✉]

[1]Key Laboratory of Pest Monitoring and Green Management, MOA; Joint Laboratory for International Cooperation in Crop Molecular Breeding; Department of Plant Pathology, China Agricultural University, Beijing 100193, China

[2]College of Plant health and medicine, Qingdao Agricultural University, Qingdao, Shandong 266109, China

[3]Environmental Sciences, University of Guelph, Guelph, Ontario, Canada

[✉]e-mail: chenxj@cau.edu.cn

This PDF file includes:

Supplementary Figures 1-25

Supplementary Tables 1-22

Supplementary References 1-6

Supplementary Figures

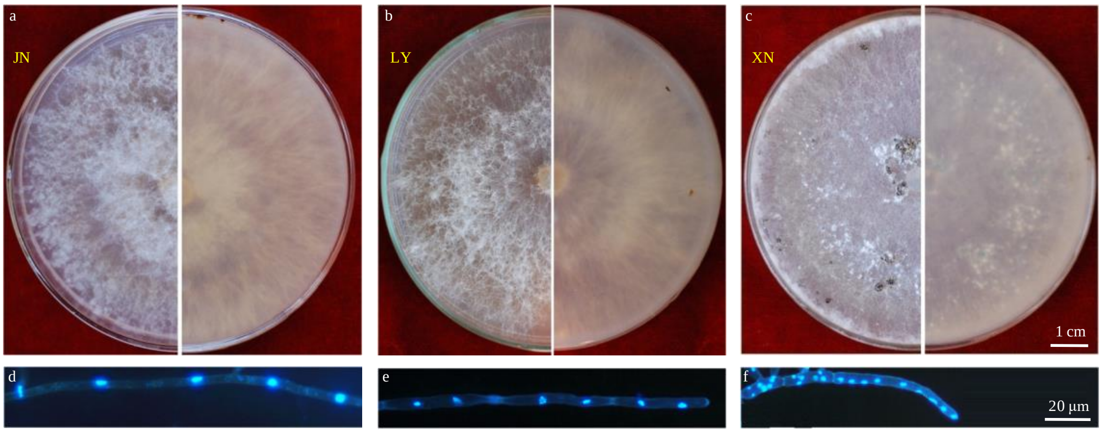

Supplementary Figure 1 *Rhizoctonia* strains JN, LY and XN. Mycelial growth of (**a**) JN, (**b**) LY, and (**c**) XN on PDA medium for 96 h and photographed from top (left) and underside (right) of each petri dish. (**d-f**) DAPI staining of nuclei in hyphae of JN (uninucleate), LY (binucleate) and XN (multinucleate).

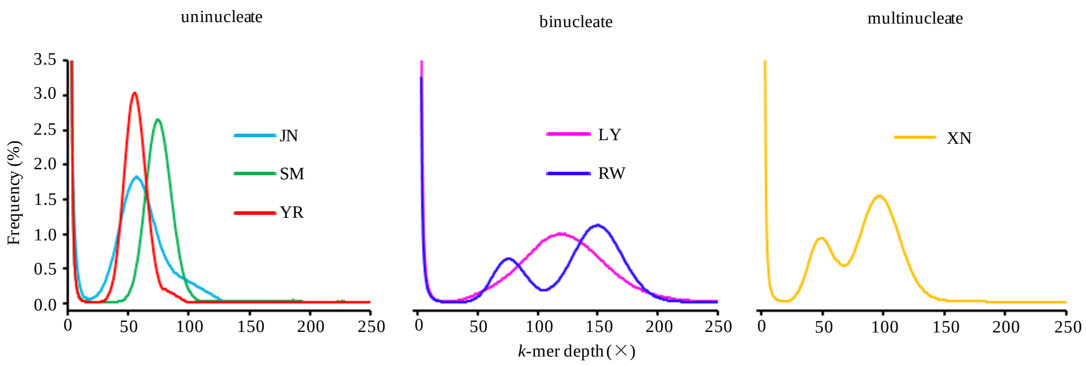

Supplementary Figure 2 The 17-mer depth distribution of whole-genome Illumina reads of *Rhizoctonia* spp.. The bimodal distributions in isolates RW and XN suggested high heterozygous genomes.

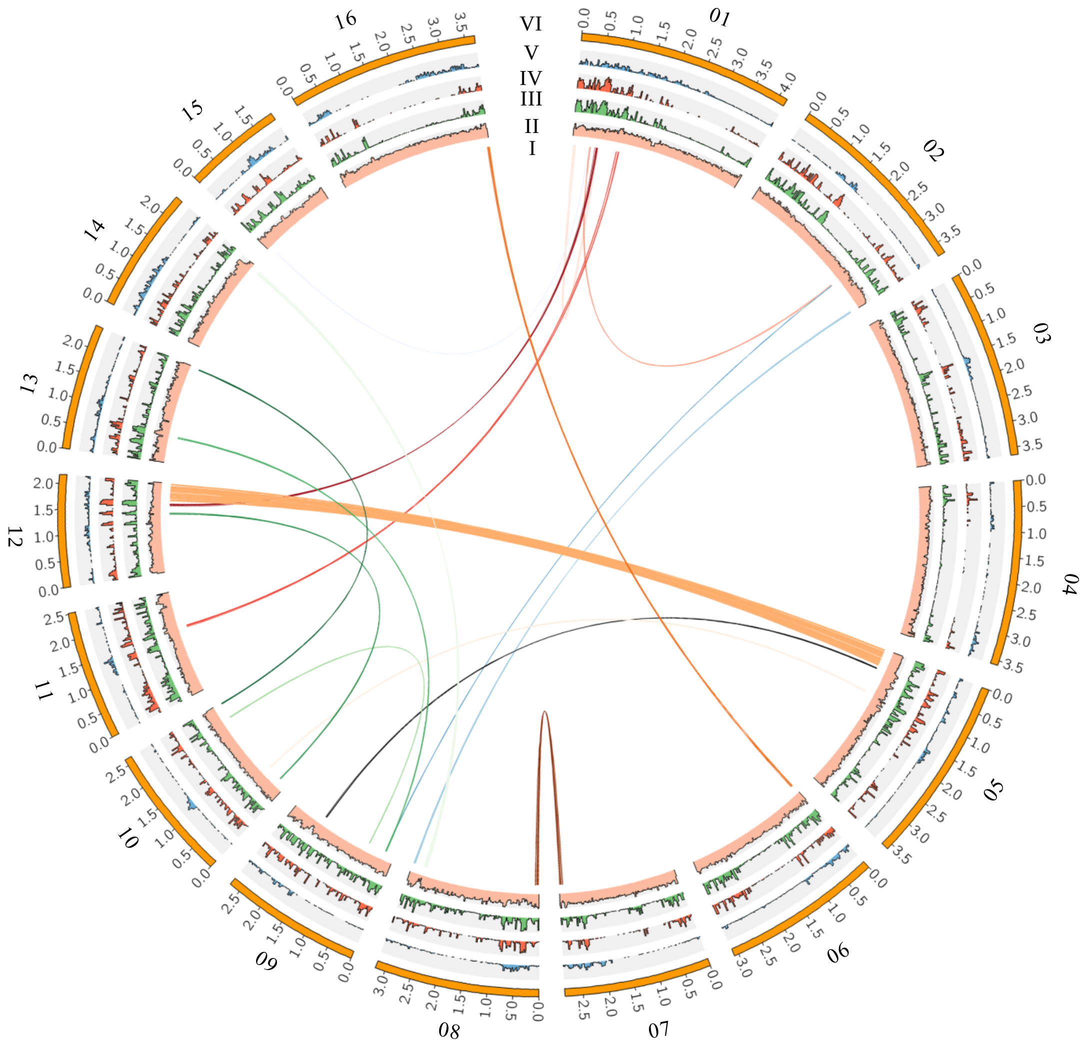

Supplementary Figure 3 Circos-plot characterizations of *Rhizoctonia* LY genome. I, the frequency distributions of read coverage (bar 0-250×). II, the syntenic gene links with 17 intragenomic syntenic fragments of 496 genes. III-V, the frequency distribution of repetitive density (bar 0-100%), LTR density (bar 0-100%) and heterozygous rate (bar 0-10‰), respectively. VI, the chromosome karyotype. All statistics were based on nonoverlapping windows (window size = 25 kb).

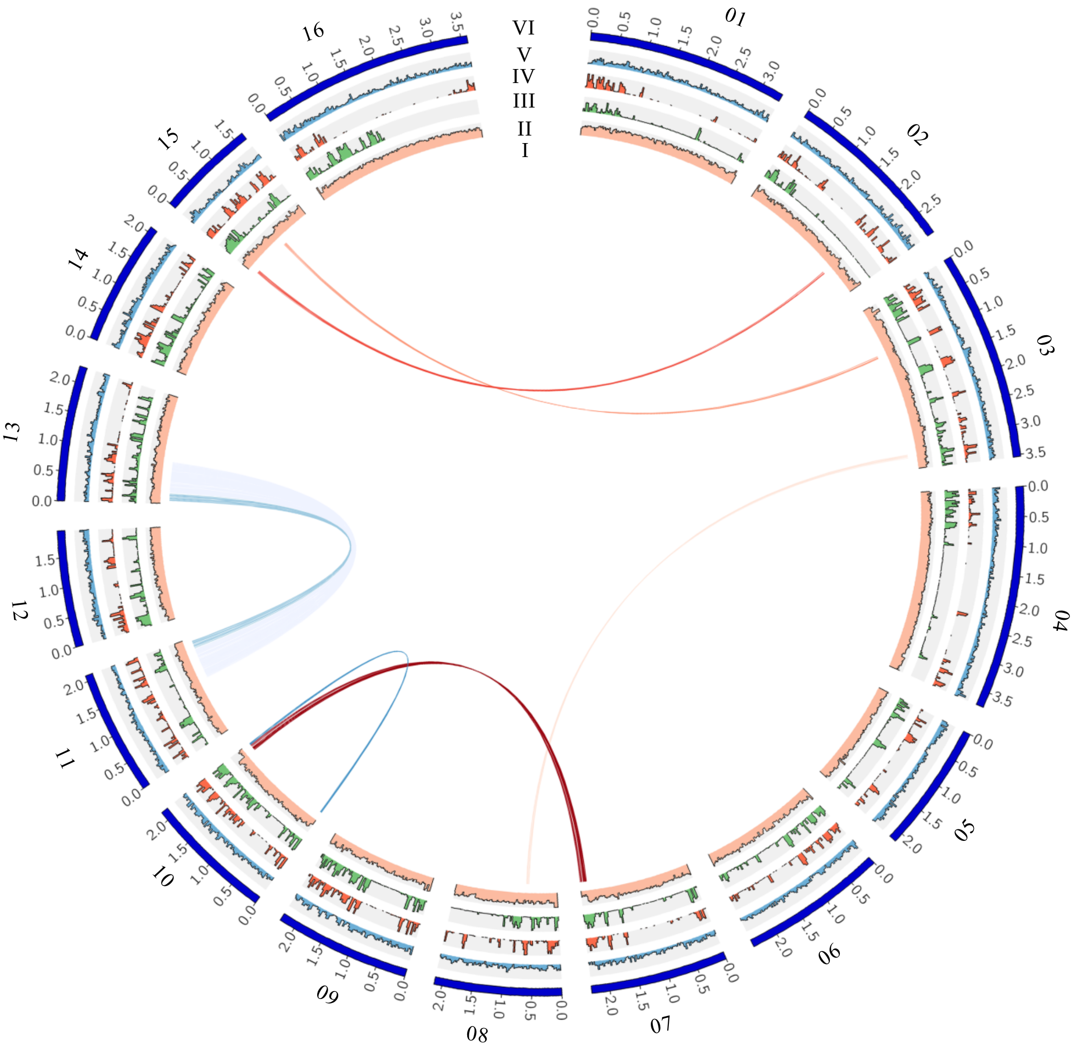

Supplementary Figure 4 Circos-plot characterization of XN genomic features. I, the frequency distributions of read coverage (bar 0-300×). II, the syntenic gene links, having eight intragenomic syntenic fragments of 464 genes. III-V, the frequency distribution of repetitive density (bar 0-100%), LTR density (bar 0-100%) and heterozygous rate (bar 0-10‰), respectively. VI, the chromosome karyotype. All statistics were based on nonoverlapping windows (window size = 25 kb).

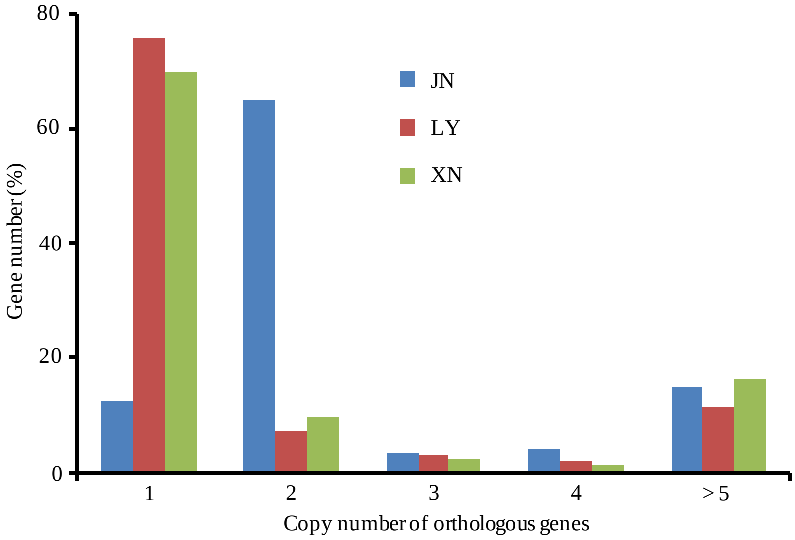

Supplementary Figure 5 Copy number distribution of orthologous genes in *Rhizoctonia* spp.. Gene number is presented as a percentage of the total genes.

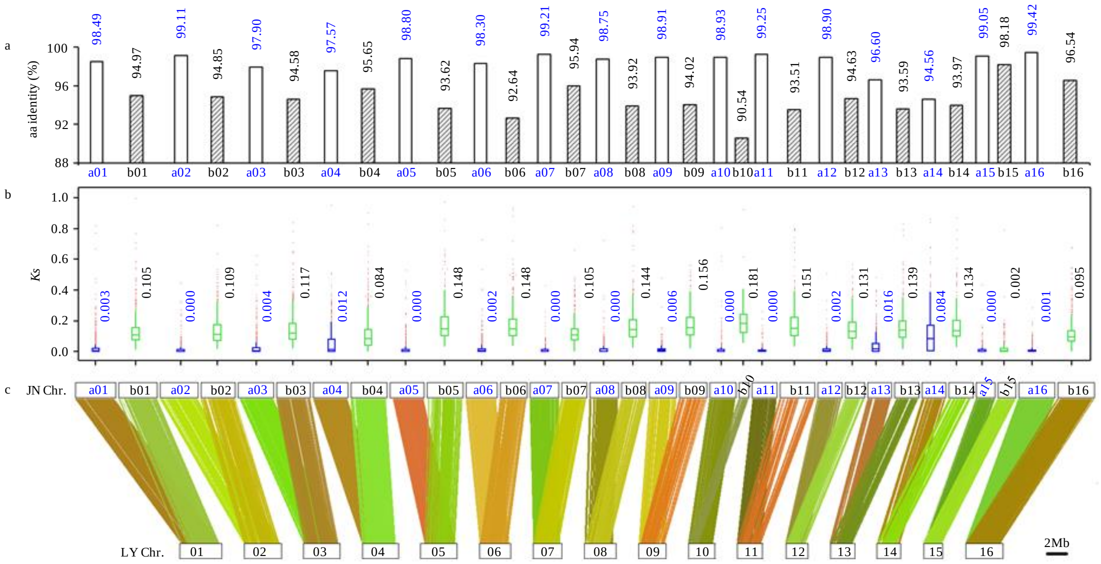

Supplementary Figure 6 Distribution of syntenic genes between JN subgenomes and LY genome. (**a**) Amino acid (aa) sequence identity between the syntenic gene pairs of LY and each JN subgenome (JNa and JNb). (**b**) Boxplots of the distribution of *Ks* between the syntenic gene pairs. (**c**) Links of syntenic genes between JN (JNa and JNb) and LY.

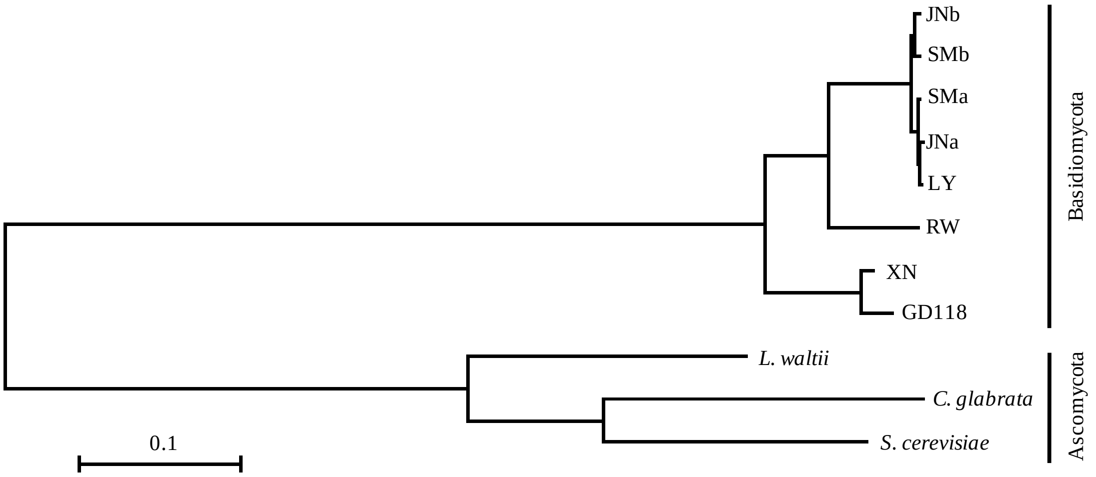

Supplementary Figure 7 Phylogenetic relationship between Basidiomycota *Rhizoctonia* and selected Ascomycota fungi using RAxML. Single-copy orthologous genes (637) were obtained for construction of the phylogenetic tree. Selected Basidiomycota *Rhizoctonia*: hybrid uninucleate JN (divided into JNa and JNb subgenomes) and SM (divided into SMa and SMb subgenomes), binucleate LY and YR, and multinucleate XN and GD118 PRJNA51401, and the reference Ascomycota: *C. glabrata* PRJNA374542, *L. waltii* PRJNA10734, *S. cerevisiae*, PRJNA183131.

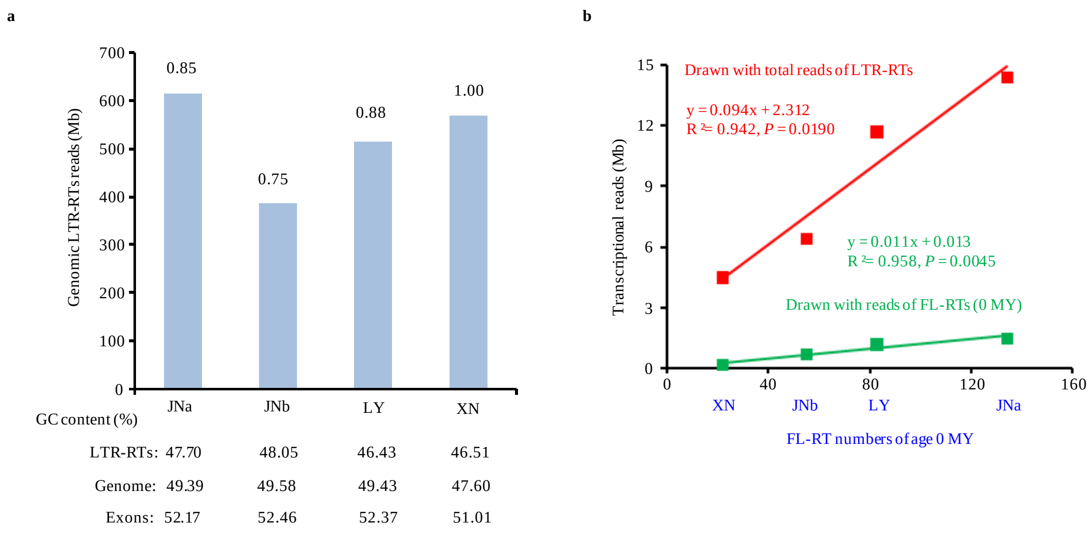

Supplementary Figure 8 Relationships of LTR-RTs with (a) genomic reads and (b) RNA-Seq reads. (**a**) The LTR-RT reads of genome sequencing were compared with the number of full-length LTR-RTs (FL-TRs). The number at the top of each bar represented the ratio of FL-TR to LTR-RT reads, normalized to XN. The bottom showed the GC content of LTR-RTs, genome and exons. (**b**) Linear relationship of transcriptional reads with the numbers of FL-RTs or FL-RTs with insertion age of 0 MY.

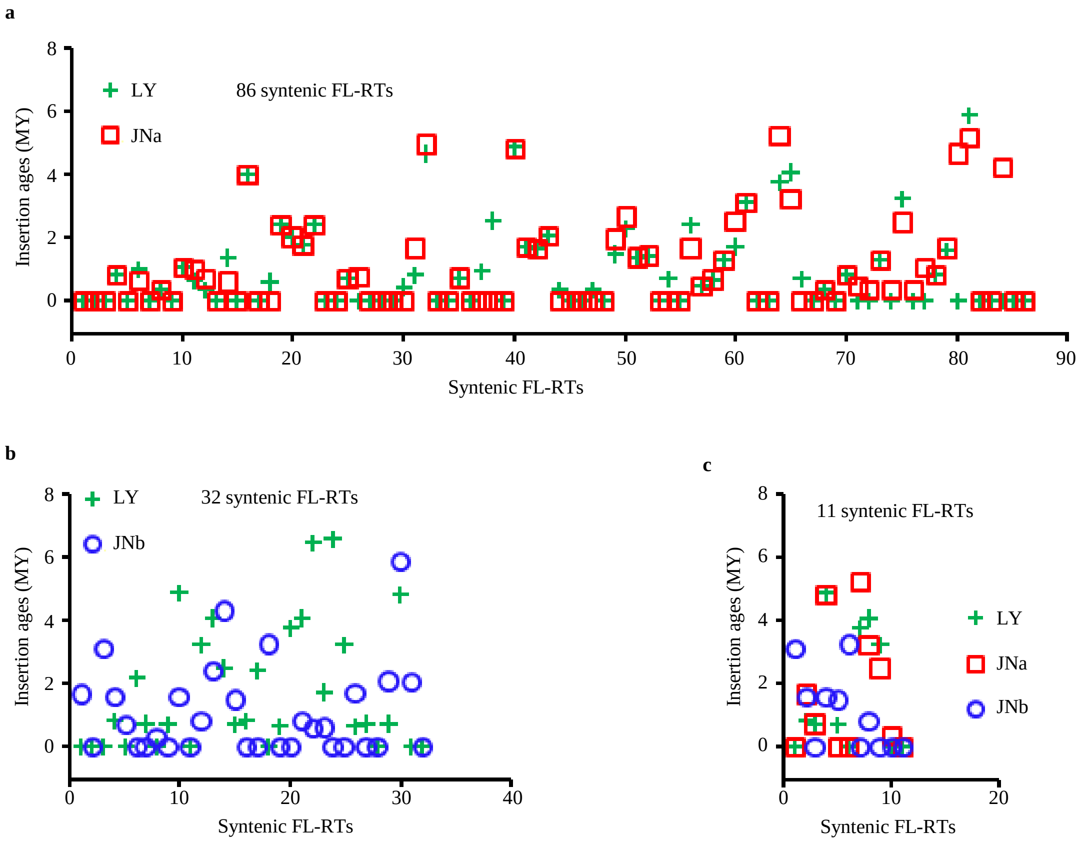

Supplementary Figure 9 Relationship of syntenic FL-RTs among LY and JN subgenomes. (**a**) between LY and JNa, (**b**) between LY and JNb, and (**c**) among JNa, JNb and LY. The FL-RTs at the same horizontal ordinate indicating synteny and the FL-RTs overlap indicating the same insertion time.

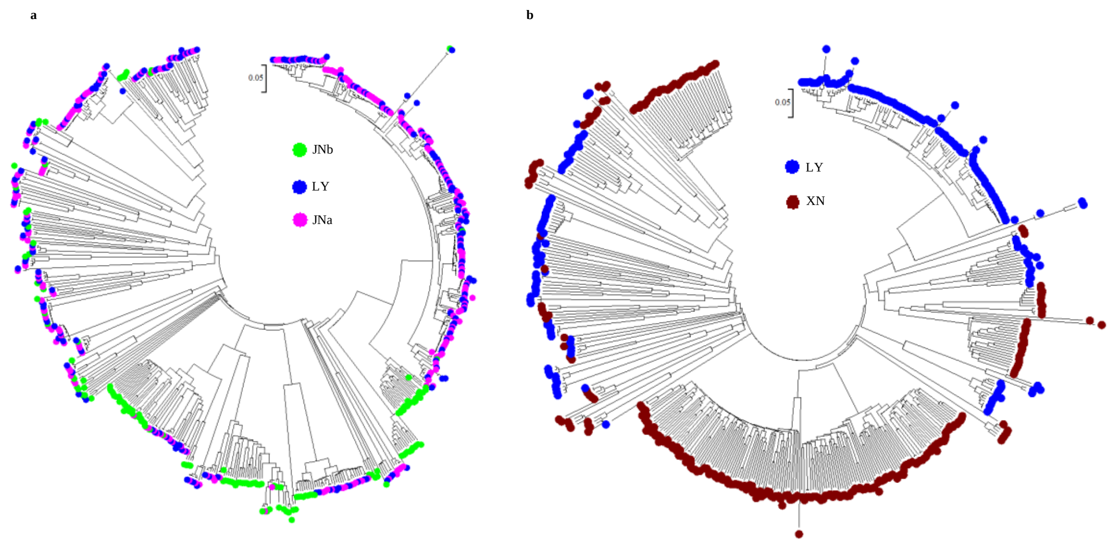

Supplementary Figure 10 Phylogenetic trees of FL-RTs for (a) JN and LY, and (b) XN and LY. FL-RT alignments were created using Clustalw and rough Neighbor-Joining trees made with MEGA6.0.

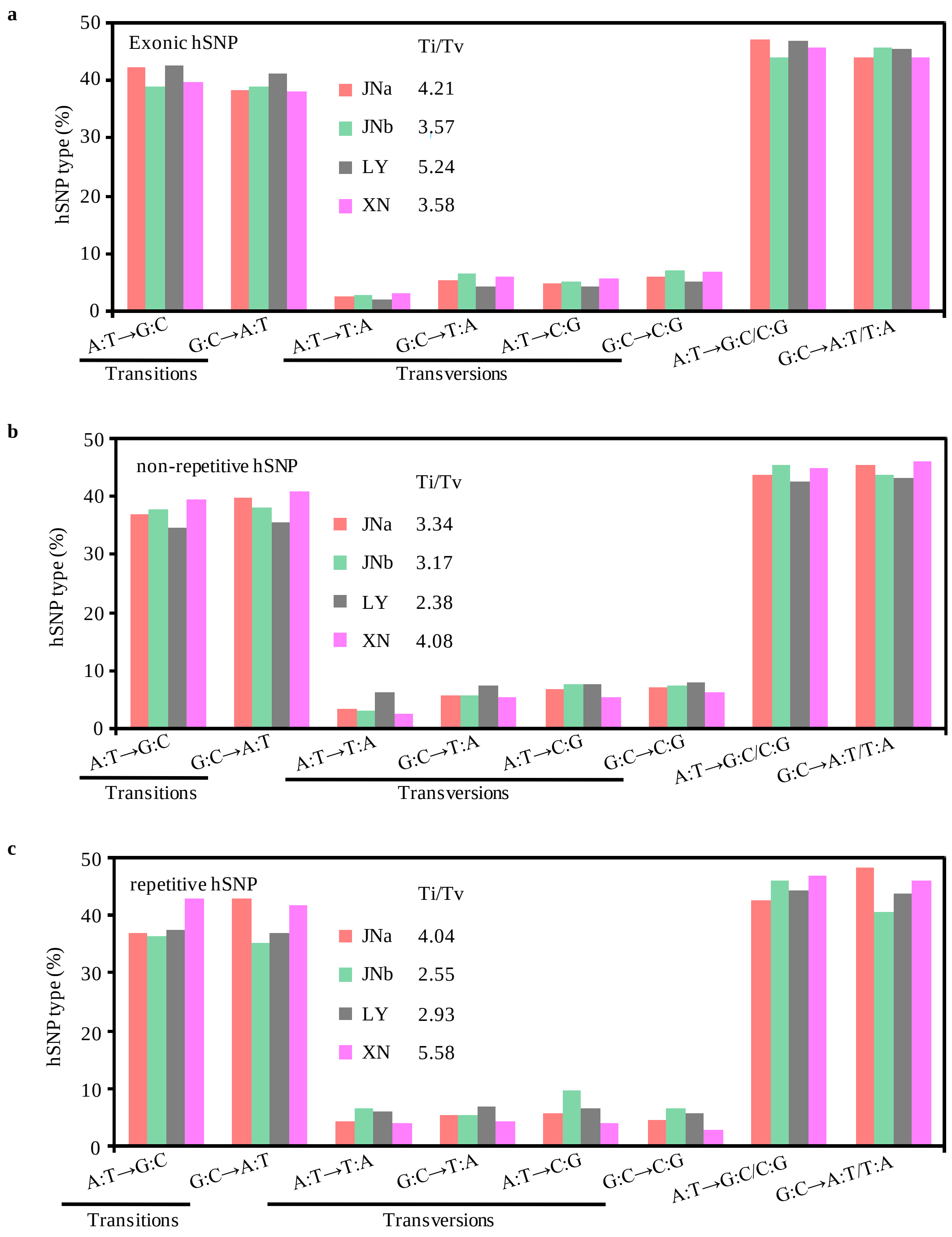

Supplementary Figure 11 Comparisons of hSNP mutational spectra in *Rhizoctonia* genomes of (a) exonic region, (b) non-repetitive region, and (c) repetitive region. Ti/Tv for the ratio of transitions to transversions.

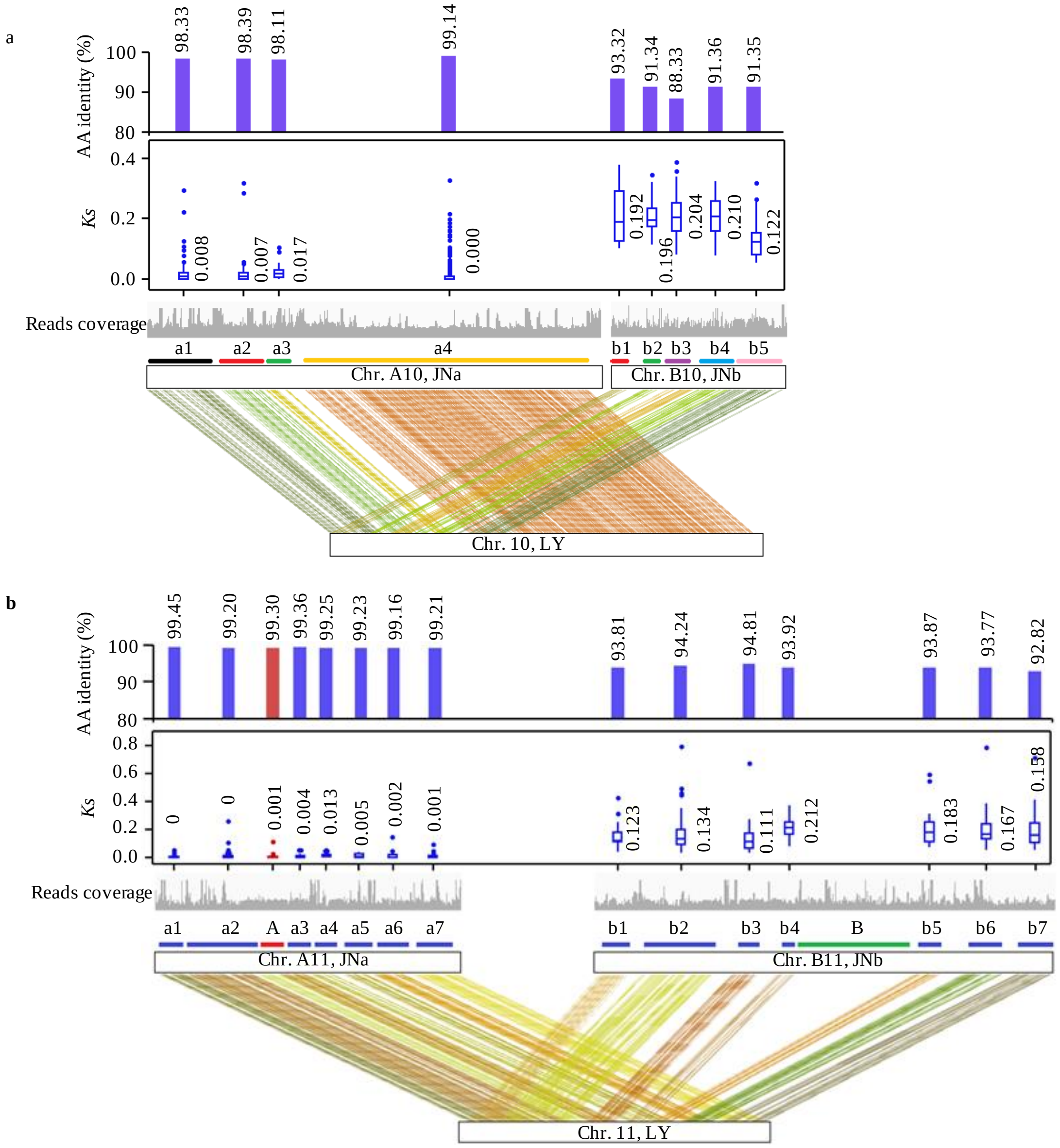

Supplementary Figure 12 Analysis of sequence insertion and deletion in JN subgenomes. (**a**) The biggest deletion in 3' end of chr. B10 and (**b**) insertion (green) in chr. B11.

a

b

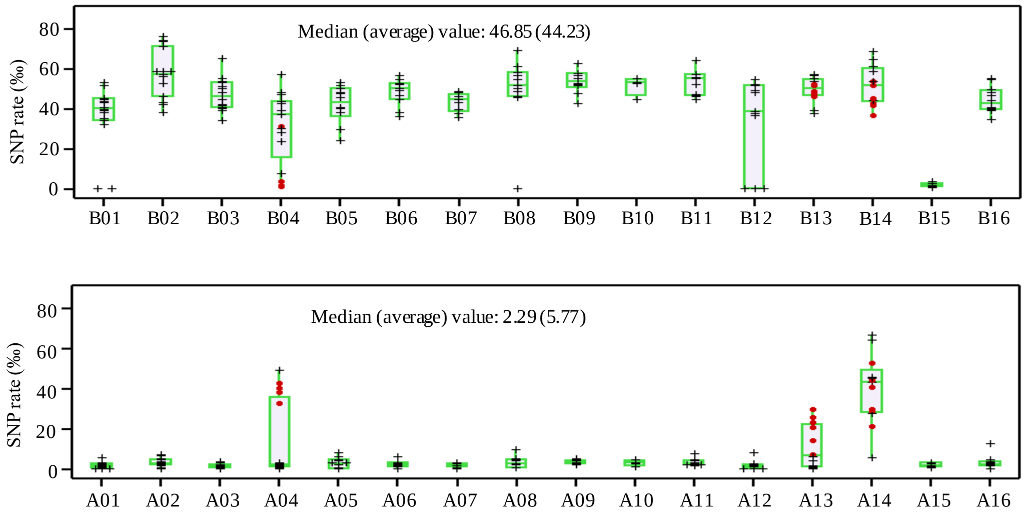

Supplementary Figure 13 SNP rates of 187 syntenic blocks between LY and (a) JNb or (b) JNa, drawn the distributions in chromosomes. Some of the blocks with gene exchanges were shown in red dots.

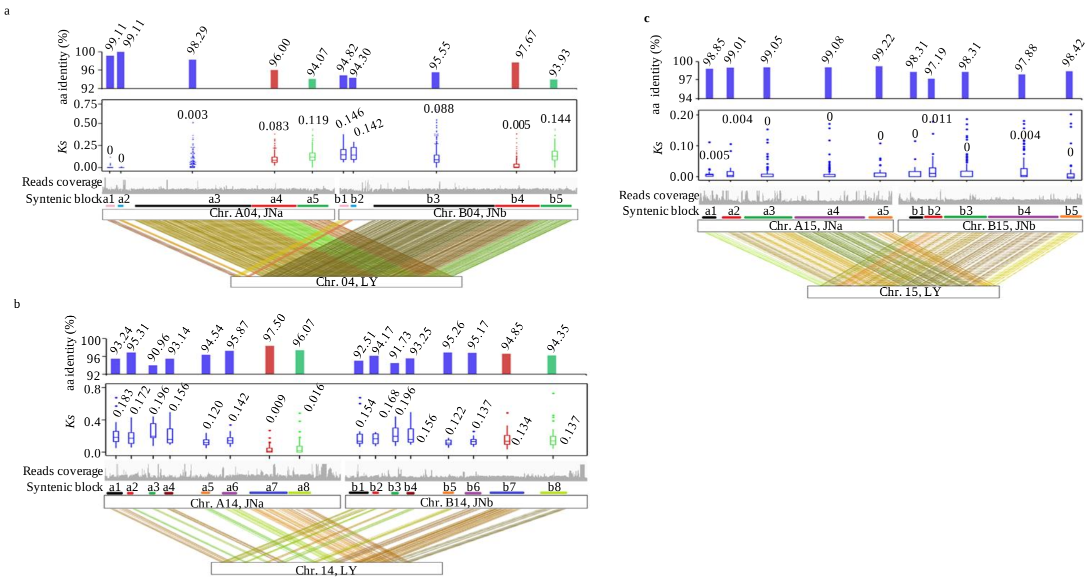

Supplementary Figure 14 Analysis of gene exchanges between JN subgenomes (JNa and JNb). The amino acid (aa) identities and *Ks* values of syntenic blocks were calculated with LY as the reference. (**a**) chr. 04, (**b**) chr. 14, and (**c**) chr. 15.

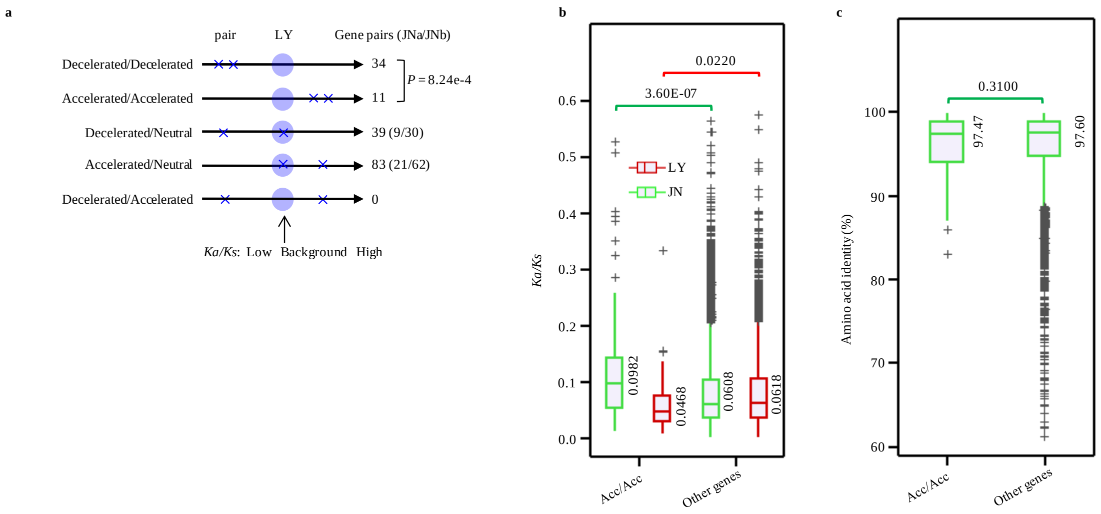

Supplementary Figure 15 Divergence of evolutionary rates and homeolog expression in JNa and JNb. (**a**) Diverged evolutionary rates of the preservation genes in the hybrid JN genome. The evolutionary rates were related to LY background with *Ka/Ks* of ±two-fold as the threshold for accelerated or decelerated evolution. *P*-values according to the binomial test. (**b**) Boxplots of *Ka/Ks* distribution and (**c**) amino acid identity between Accelerated/Accelerated (Acc/Acc) and the rest of gene pairs (Other genes). *P*-values according to the "Mann-Whitney U test".

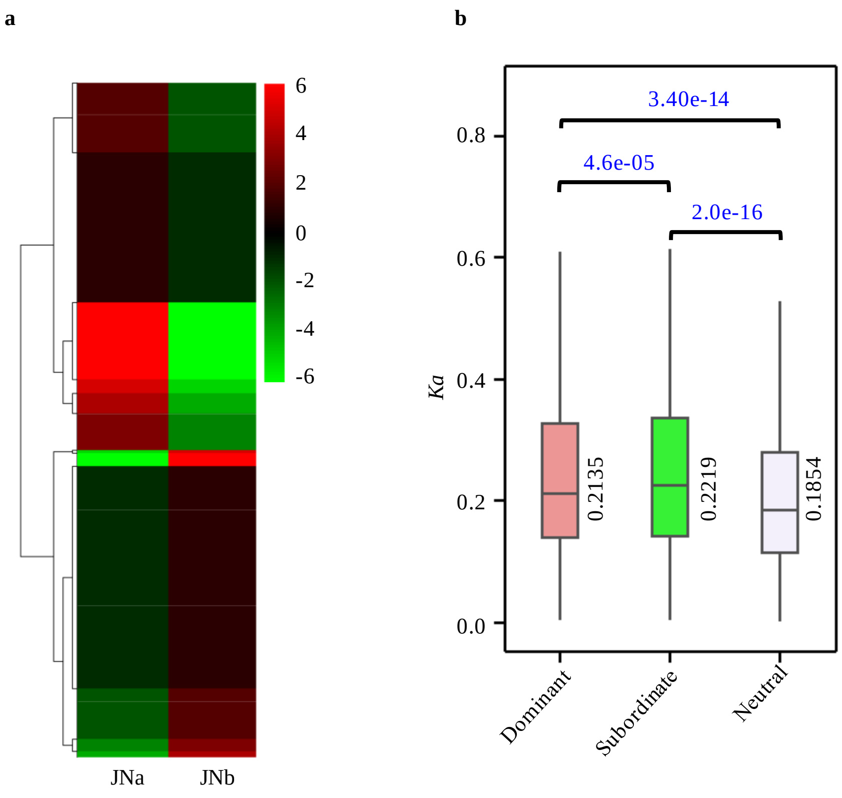

Supplementary Figure 16 Heatmap showing genome-wide expression of syntenic homeolog gene pairs between JNa and JNb. (**a**) Mycelium of JN was grown in the potato dextrose culture 48 h and used for transcriptomic analysis. (**b**) Boxplot showing the distribution of *Ka* values among homeolog expression dominance genes as dominant, subordinate and neutral in JN. Significant differences were analyzed using the "Mann-Whitney U test".

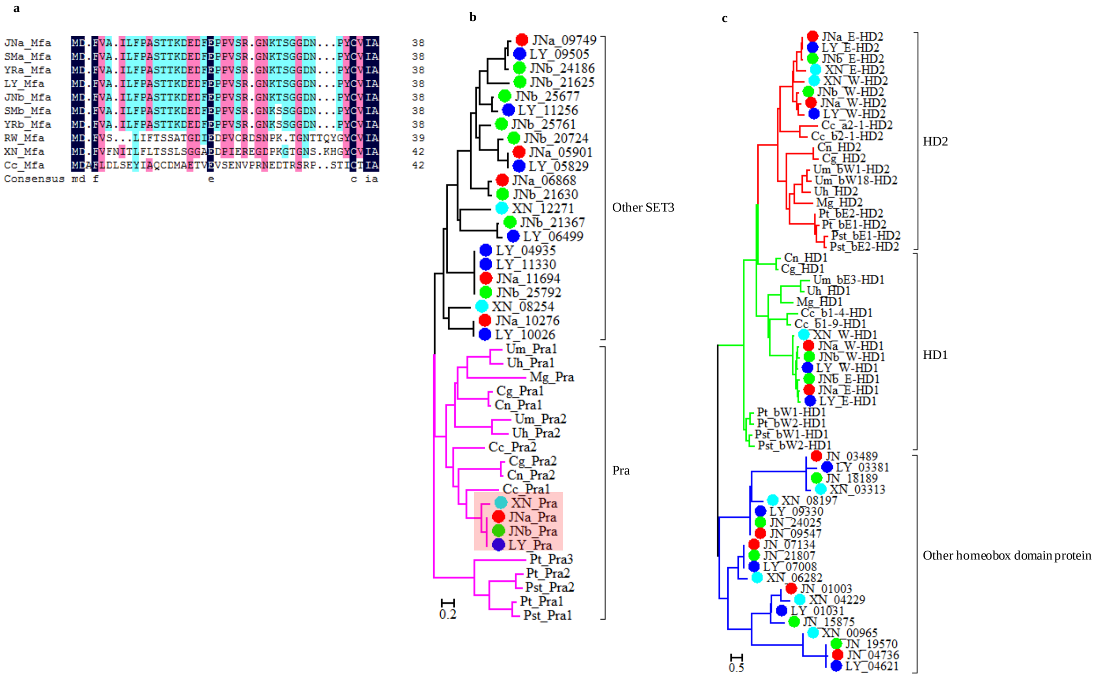

Supplementary Figure 17 Comparisons of mating loci of *Rhizoctonia* with selected Basidiomycota fungi. (**a**) Sequence alignment of *Rhizoctonia* Mfa. Pheromone (Mfa) proteins were collected and shown as species _protein name (accession No.): JNa_Mfa (chr. 15A: 993630-749), JNb_Mfa (chr. 15B: 929651-770), SMa_Mfa (scaffold034: 404386-505), SMb_Mfa (scaffold013: 1096589-708), YRa_Mfa (scaffold033: 115988-6107), YRb_Mfa (scaffold085: 70200-319), LY_Mfa (chr.15: 956133-252), RW_Mfa (scaffold0051: 694268-390), XN_Mfa (chr15: 699219-350), *C. cinerea* (Cc_Mfa, XP_002910173). Phylogenetic relationships of *Rhizoctonia* Pra (**b**) and HD (**c**) with some selected Basidiomycota fungi. Pheromone receptor Pra or other SET3-like proteins were collected and shown as species_protein name (accession No.): JNa_Pra (JN_13413), JNb_Pra (JN_27476), LY_Pra (LY_12967), XN_Pra (XN_12028), *C. cinerea* (Cc_Pra1, AAF01418; Cc_Pra2, AAQ96344), *C. neoformans* (Cn_Pra1, AAN75624; Cn_Pra2, AAN75724), *P. triticina* (Pst_Pra1, KNE91511; Pst_Pra2, KNE91469), *U. maydis* (Um_Pra1, AAA99766; Um_Pra2, AAA99768), *P. triticina* (Pt_Pra1, OAV89066; Pt_Pra2,

OAV87231; Pt_Pra3, OAV88334), *U. hordei* (Uh_Pra1, CAJ41875; Uh_Pra2, AAD56044), *C. gattii* (Cg_Pra1, AEG78597; Cg_Pra2, XP_003196044), *M. globosa* (Mg_Pra, EDP44482) and other SET3-like protein (JN_05901, JN_06868, JN_09749, JN_10276, JN_11694, JN_20724, JN_21367, JN_21625, JN_21630, JN_24186, JN_25677, JN_25761, JN_25792, LY_04935, LY_05829, LY_06499, LY_09505, LY_10026, LY_11256, LY_11330, XN_08254, XN_12271). HD proteins or other homeobox domain: JNa_W-HD2 (JN_11348), JNa_W-HD1 (JN_11349), JNa_E-HD1 (JN_11350), JNa_E-HD2 (JN_11351), JNb_W-HD2 (JN_25554), JNb_W-HD1 (JN_25555), JNb_E-HD1 (JN_25556), JNb_E-HD2 (JN_25557), LY_W-HD2 (LY_11067), LY_W-HD1 (LY_11068), LY_E-HD1 (LY_11069), LY_E-HD2 (LY_11070), XN_E-HD2 (XN_11532), XN_W-HD1 (XN_11535), XN_W-HD2 (XN_11536), *C. cinerea* (Cc_a2-1-HD2, CAA56131; Cc_b1-4-HD1, AAD33326; Cc_b1-9-HD1, AAD33323; Cc_b2-1-HD2, CAA56132), *C. neoformanns* (Cn_HD1, AAN75718; Cn_HD2, AAV98474), *P. striiformis* (Pst_bE1-HD2, Pst_bW1-HD1, Pst_bW2-HD1 and Pst_bE2-HD2), *U. maydis* (Um_bE3-HD1, P22017; Um_bW1_HD2, XP_756725; Um_bW18_HD2, CAF34004), *P. triticina*[1] (Pt_bW1-HD1, Pt_bW2-HD1, Pt_bE1-HD2 and Pt_bE2-HD2), *C. gattii* (Cg_HD1, AAV28797; Cg_HD2, AAZ07733), *U. hordei* (Uh_HD1, CAA79218; Uh_HD2, CAA79219), *M. globosa* (Mg_HD1, EDP44402; Mg_HD2, EDP44401) and other homeobox domain protein (JN_01003, JN_03489, JN_04736, JN_07134, JN_09547, JN_15875, JN_18189, JN_19570, JN_21807, JN_24025, LY_01031, LY_03381, LY_04621, LY_07008, LY_09330, XN_00965, XN_03313, XN_04229, XN_06282 and XN_08197). Phylogenetic trees of mating-type proteins were constructed using RAxML.

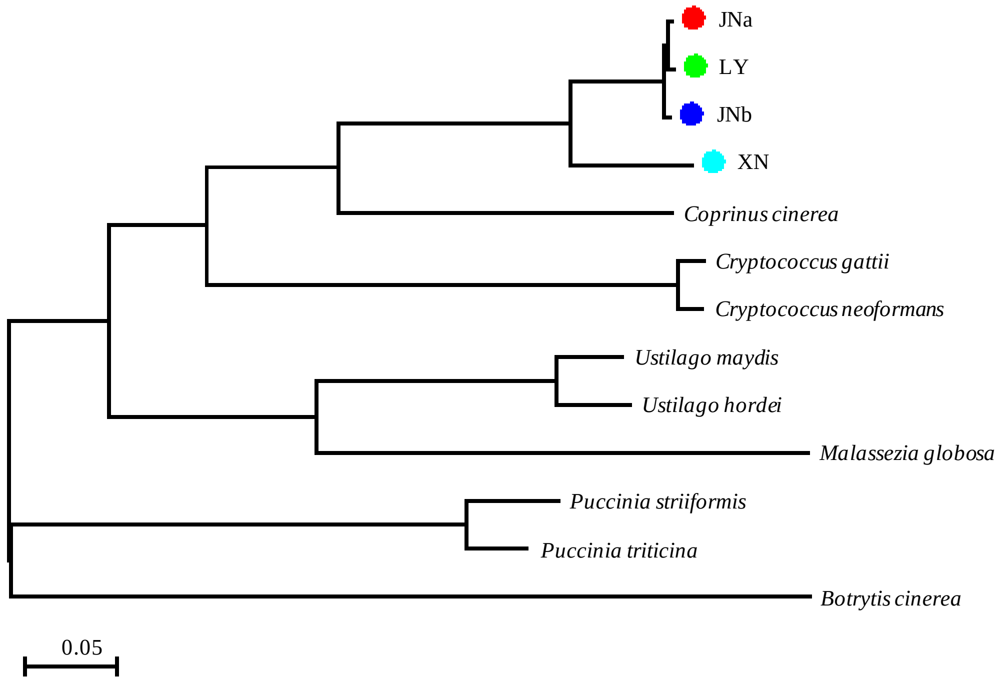

Supplementary Figure 18 Phylogenetic tree was constructed using RAxML from 889 single-copy ortholog genes for *Rhizoctonia* and other fungi. *Rhizoctonia*: JN (divided into JNa and JNb subgenomes), LY, and XN. Other fungi: *Coprinus cinerea* No. PRJNA1447, *Cryptococcus gattii* No. PRJNA13692, *C. neoformans* No. PRJNA411, *Ustilago maydis* No. PRJNA1446, *U. hordei* No. PRJEA79049, *Malassezia globosa* No. PRJNA18719, *Puccinia striiformis* No. PRJNA396589, *P. triticina* No. PRJNA36323, and *Botrytis cinerea* No. PRJNA15632.

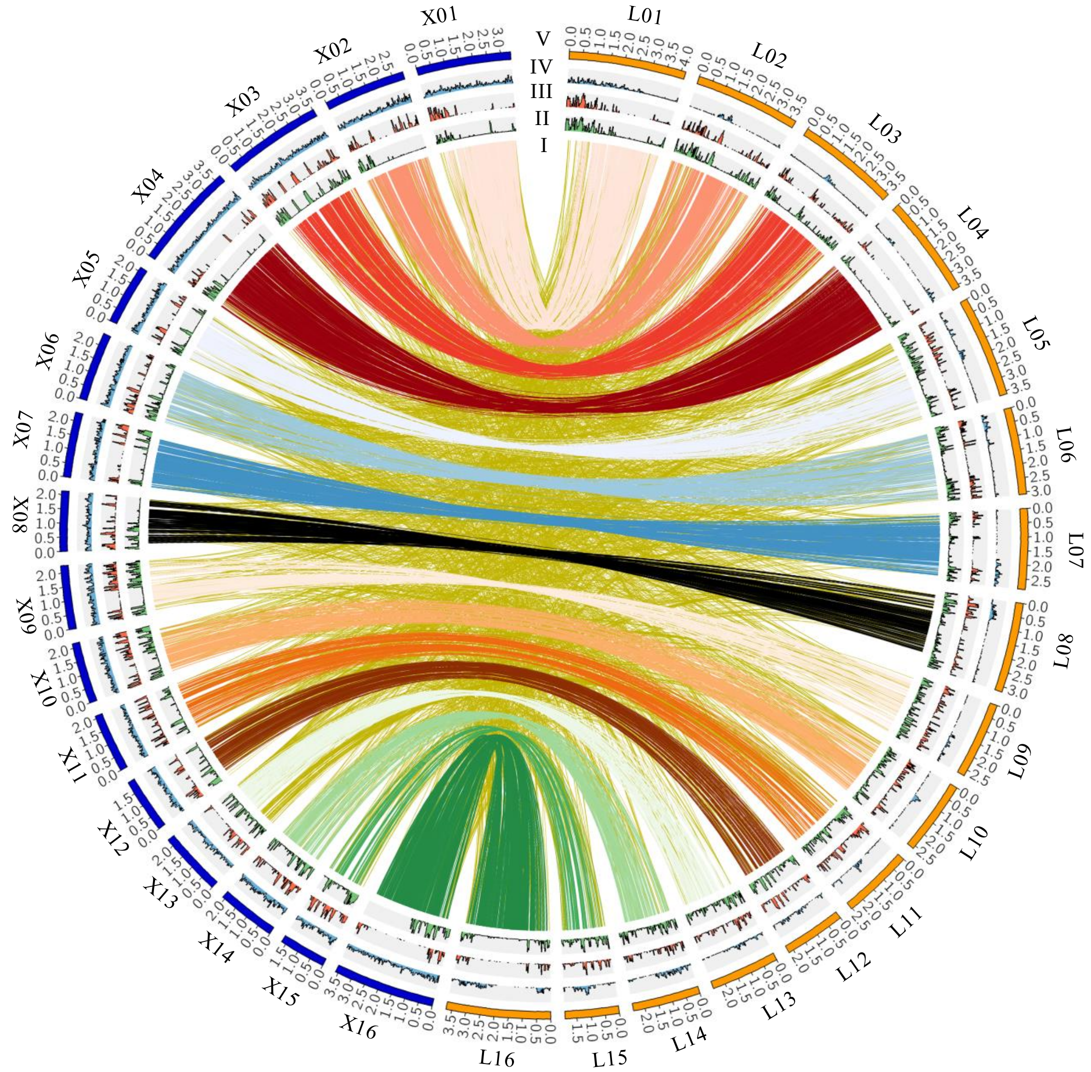

Supplementary Figure 19 Circos-plot characterization of genome features of LY and XN. I, represents the homologous gene links (blastp, coverage ≥50, identity ≥50 and E-value ≤1e-15). II-IV, the frequency distribution of repeat density (bar 0-100%), LTR density (bar 0-100%) and heterozygous rate (bar 0-10‰). V, indicating the chromosome karyotype (prefix L for LY and X for XN) . All statistics were based on nonoverlapping windows (window size = 25 kb).

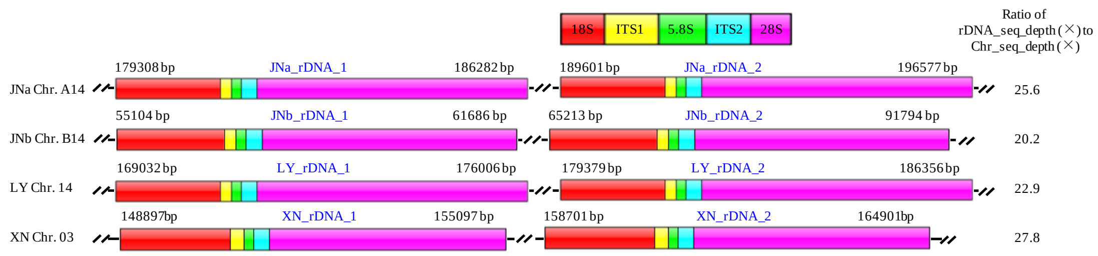

Supplementary Figure 20 Organization of rDNA locus in *Rhizoctonia* spp.. The rDNA repeats were calculated as the ratio of rDNA_seq_depth (×) to Chr_seq_depth (×) .

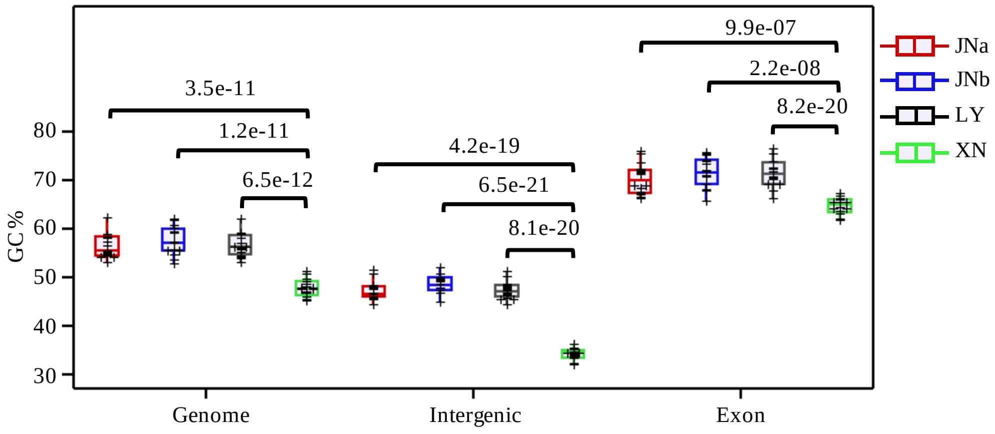

Supplementary Figure 21 Comparison of genome-wide GC contents among JNa, JNb, LY, and XN. *P*-values shown in corresponding comparisons.

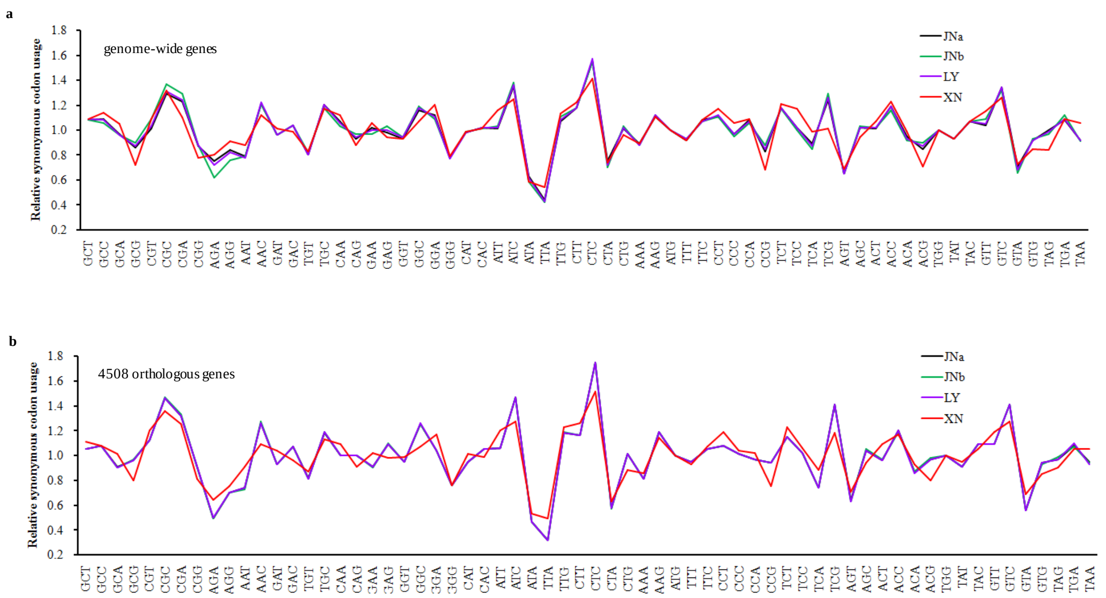

Supplementary Figure 22 Relative synonymous codon usage of genes of (a) genome-wide and (b) 4508 orthologs among JNa, JNb, LY, and XN.

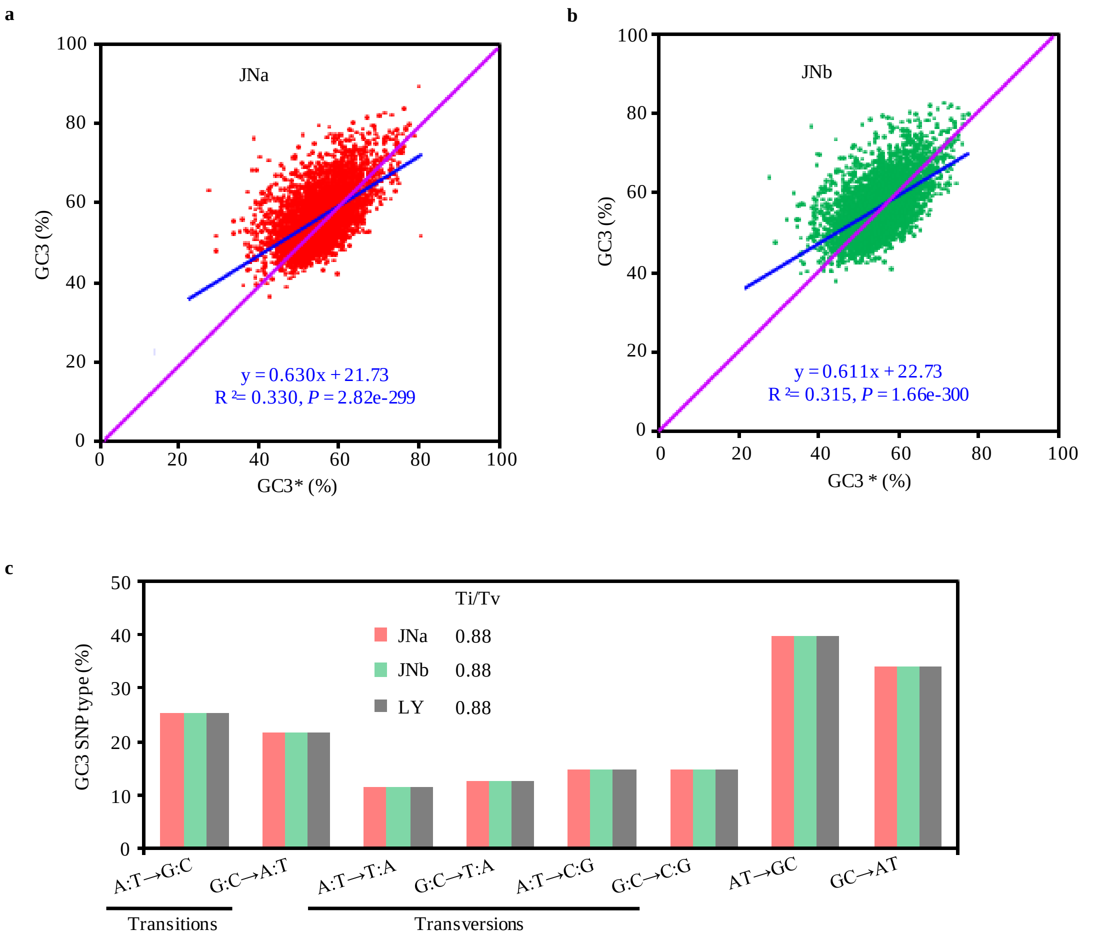

Supplementary Figure 23 Comparison of GC contents of 4508 orthologous genes among JNa, JNb, LY, and XN. (**a-b**) Correlation between the frequency of GC3 and GC3*, reflecting the AT to GC substitution rates of JN subgenomes (JNa and JNb) in relation to XN orthologs at the third codon positions. (**c**) GC3 SNP mutation patterns and ratios of transition (Ti) to transversion (Tv) in the data sets.

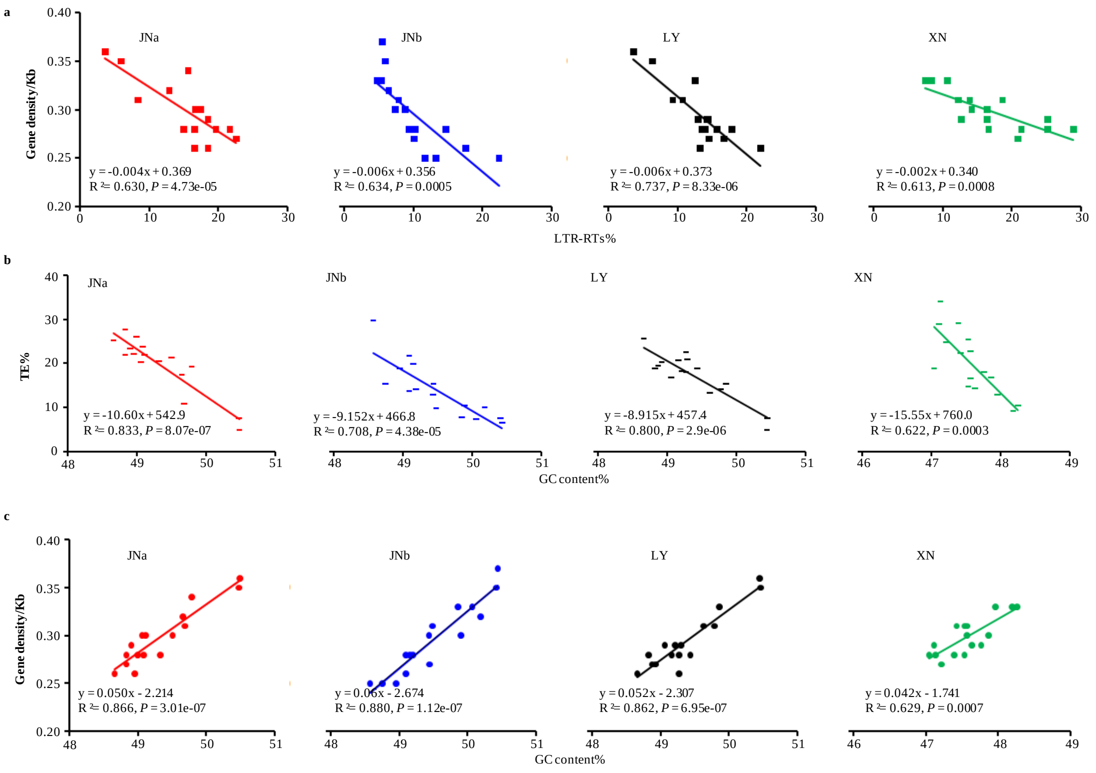

Supplementary Figure 24 Correlation of LTR-RTs or TE with gene density and GC content in *Rhizoctonia*. (**a**) Correlation between the gene density and LTR-RTs%. (**b**) Correlation between the TE% and GC content. (**c**) Correlation between the gene density and GC%.

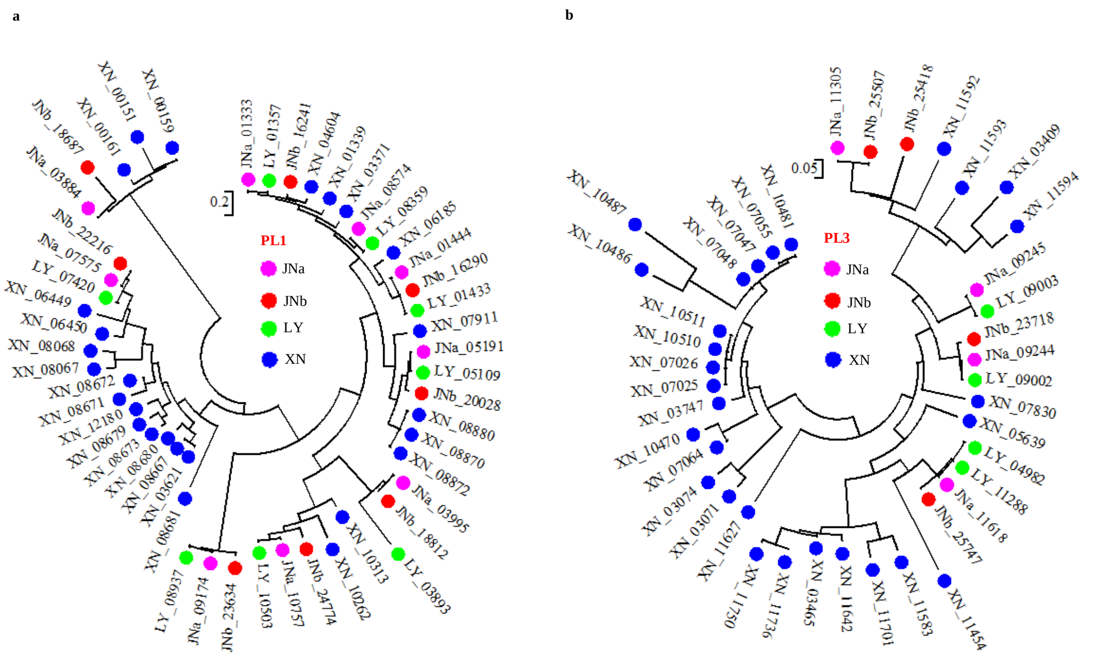

Supplementary Figure 25 Phylogenetic tree of polysaccharide lyases (PLs, PL1 and PL3) genes from *Rhizoctonia* JN (divided into JNa and JNb subgenomes), LY, and XN using RAxML.

Supplementary Tables

Supplementary Table 1 Illumina reads used in the assembly of *Rhizoctonia* spp.

Nuclear status	ID	Insert size	Raw reads			Filter reads		
			Bases (Mb)	GC%	#	Bases (Mb)	GC%	#
Uninucleate	JN	180 bp	2,434	47.22	22	2,170	46.94	19
		500 bp	6,121	47.35	54	5,171	46.94	46
		2 kb	2,881	47.79	26	1,560	47.37	14
		5 kb	1,625	47.95	14	1,047	47.69	9
		10 kb	3,959	47.51	35	2,868	47.43	25
		Total	17,020	47.56	151	12,815	47.27	114
	SM	350 bp	8,962	49.09	97	8,701	49.07	94
		2 kb	5,626	47.45	61	5,291	47.12	57
		5 kb	5,583	47.6	60	5,364	47.23	58
		Total	20,171	48.05	217	19,356	47.81	209
	YR	350 bp	6,598	49.39	69	6,439	49.38	67
		2 kb	6,622	47.45	69	6,622	47.47	69
		5 kb	5,019	47.52	52	4,983	47.53	52
		Total	18,239	48.12	191	18,044	48.13	189
Binucleate	LY	500 bp	8,467	47.16	152	7,386	46.82	132
		2 kb	1,541	47.5	28	1,090	47.15	20
		5 kb	1,034	48.15	19	684	47.79	12
		Total	11,042	47.6	198	9,160	47.25	164
	RW	350	4,835	48.82	117	4,606	48.79	112
		2 kb	3,843	47.35	93	3,434	47.32	83
		5 kb	3,141	47.1	76	2,882	47.11	70
		Total	11,820	47.76	287	10,922	47.74	265
Multinucleate	XN	500 bp	5,429	45.66	121	4,537	45.22	101
		2 kb	1,925	46.5	43	1,530	46.84	34
		5 kb	1,073	46.31	24	803	46.77	18
		Total	8,428	46.16	188	6,870	46.28	154

representing genome coverage times (×)

Supplementary Table 2 Description of *Rhizoctonia* genome assembled using Illumina reads

Nuclear status	ID	Scaffold features					
		Size (bp)	Count	N50 (bp)	Longest[1]	Average[1]	Genes
Uninucleate	JN[2]	93,419,130	360	1,037,862	3,969,832	259,498	27,397
	JNa	46,213,895	146	478,788	2,054,009	316,534	13,575
	JNb	45,026,776	39	1,593,039	3,969,832	1,154,533	13,328
	SM[3]	92,745,416	547	706,404	3,757,952	169,553	25,636
	SMa	44,699,832	274	298,430	1,905,312	163,138	12,493
	SMb	46,320,242	62	1,579,645	3,757,952	747,101	12,810
	YR[4]	95,662,546	628	569,039	3,600,218	152,329	26,384
	YRa	48,122,350	350	314,472	2,433,256	137,492	13,308
	YRb	47,143,522	181	956,173	3,600,218	260,461	11,645
Binucleate	LY	47,655,842	668	252,999	2,469,184	71,341	14,079
	RW	45,920,232	2,506	302,546	1,932,229	18,324	13,453
Multinucleate	XN	40,972,687	956	105,466	458,737	42,858	12,166

[1]Representing the Longest or Average length of scaffold (bp)

[2]Divided into JNa and JNb subgenomes; 175 scaffolds (2,178,459 bp, 494 genes) of JN could not be assigned to the JNa and JNb subgenoemes.

[3]Divided into SMa and SMb subgenomes; 211 scaffolds (1,725,342 bp, 333 genes) of SM could not be assigned to the SMa and SMb subgenoemes.

[4]Divided into YRa and YRb subgenomes; 97 scaffolds (396,674 bp, 71 genes) of YR could not be assigned to the YRa and YRb subgenoemes.

Supplementary Table 3 PacBio reads of *Rhizoctonia* spp. for assembling

Strain	Term	Raw reads	Filtered reads
JN	Total size (Mb)	14,358	11,267
	Sequence count	1,379,545	641,836
	Longest length (bp)	78,101	78,101
	Average length (bp)	10,408	17,555
	GC content	49.61%	49.63%
	Genome coverage (×)	147	116
LY	Total size (Mb)	15,173	11,146
	Sequence count	1,660,614	639,809
	Longest length (bp)	77,290	77,290
	Average length (bp)	9,137	17,421
	GC content	49.41%	49.44%
	Genome coverage (×)	309	227
XN	Total size (Mb)	14,397	11,478
	Sequence count	1,364,440	992,996
	Longest length (bp)	77,154	77,154
	Average length (bp)	9,086	11,559
	GC content	47.80%	47.80%
	Genome coverage (×)	344	275

Supplementary Table 4 Estimation of genome size by 17-mer statistics

Strain	*k*-mer	*k*-mer_num	*k*-mer_depth	Estimated size (bp)
JN	17	5,648,785,126	58	97,392,847
LY	17	6,184,521,252	126	49,083,502
XN	17	4,054,655,193	97	41,800,569

The assembling size of each genome is over 98% its content estimated by *k*-mer analysis.

Supplementary Table 5 Characteristics of JN chromosomes

ChrID*	Size/bp	GC%	Genes	Repeat%	LTR%	SNP‰	Heterozygosity‰
ChrA01	4,178,437	49.79	1403	21.76	15.59	1.76	0.04
ChrA02	3,921,936	49.11	1176	26.35	17.39	2.08	0.07
ChrA03	3,698,223	49.68	1133	13.33	8.32	1.41	0.07
ChrA04	3,609,862	50.48	1309	6.58	3.67	15.51	1.29
ChrA05	3,559,238	48.90	1017	27.38	18.43	1.51	0.1
ChrA06	3,231,384	48.83	909	26.3	16.56	1.47	0.11
ChrA07	2,995,745	49.65	949	19.94	12.85	1.7	0.1
ChrA08	2,951,873	49.50	883	25.45	16.58	2.36	0.11
ChrA09	2,870,714	48.83	773	32.18	22.51	3.59	0.09
ChrA10	2,763,397	49.08	776	27.8	19.6	2.11	0.03
ChrA11	2,584,362	48.66	676	28.85	18.45	2.35	0.07
ChrA12	2,575,755	48.99	715	30.61	21.58	1.5	0.15
ChrA13	2,454,648	49.06	745	23.97	17.03	1.58	0.1
ChrA14#	2,464,062	49.32	681	25.74	14.96	27.4	0.99
ChrA15	1,976,081	48.95	519	26.57	16.49	1.92	0.03
ChrA16	3,729,354	50.47	1314	9.81	5.96	2.07	0.12
ChrB01	4,014,316	50.18	1278	12.52	6.44	43.5	0.02
ChrB02	3,531,623	49.48	1081	13.28	7.88	53.56	0.01
ChrB03	3,505,788	49.85	1140	10.27	5.45	50.46	0.02
ChrB04	3,716,352	50.43	1374	8.29	5.47	32.29	1.04
ChrB05	3,737,021	49.44	1006	19.2	10.12	55.64	0.01
ChrB06	2,791,466	49.09	791	16.26	9.27	60.49	0.01
ChrB07	2,608,004	50.06	858	9.99	4.82	49.46	0.01
ChrB08	2,539,657	49.43	770	15.89	8.83	59.61	0.01
ChrB09	2,857,125	49.09	752	25.63	17.47	58.42	0.01
ChrB10	1,062,074	48.75	261	19.93	11.64	70.88	0.03
ChrB11	3,599,671	48.57	901	34.44	22.29	56.44	0.02
ChrB12	2,061,578	49.89	614	13.72	7.37	42.93	0.02
ChrB13	2,514,649	49.19	713	17.17	10.27	57.55	0.27
ChrB14#	2,513,835	49.15	713	24.51	14.68	58.05	0.91
ChrB15	1,857,660	48.95	467	23.16	13.22	2.55	0.12
ChrB16	3,814,631	50.41	1324	9.77	5.9	45.41	0.02

*telomeric sequence 5'-TTAGG-3' at the 3'-terminus and 5'-CCTAA-3' at the 5'-terminus ends of nuclear chromosomes.

#rDNA repeats presence

Supplementary Table 6 Characteristics of LY chromosomes

ChrID[*]	Size(bp)	GC%	Genes	Repeat%	LTR%	Heterozygosity‰
Chr01	4,163,667	49.85	1389	18.96	12.49	0.90
Chr02	3,730,069	49.21	1100	22.46	14.44	0.33
Chr03	3,645,922	49.62	1123	15.82	10.73	0.42
Chr04	3,589,637	50.44	1299	6.61	3.62	0.39
Chr05	3,622,931	49.06	1066	23.58	12.95	0.52
Chr06	3,089,330	48.82	851	23.09	14.03	0.72
Chr07	2,855,537	49.78	876	17.31	9.29	0.55
Chr08	3,192,901	49.43	897	23.35	13.47	0.60
Chr09	2,723,000	48.92	733	28.23	16.69	0.41
Chr10	2,623,369	49.16	742	24.39	15.69	0.38
Chr11	2,515,494	48.66	661	32.61	21.93	0.48
Chr12	2,177,546	49.27	618	28.63	17.82	0.74
Chr13	2,441,977	49.29	718	26.69	14.18	0.67
Chr14[#]	2,408,520	49.27	635	23.23	13.27	1.11
Chr15	1,926,103	48.87	511	25.13	14.47	1.01
Chr16	3,695,994	50.45	1297	10.57	6.36	0.77

[*]telomeric sequence 5'-TTAGG-3' at the 3'-terminus and 5'-CCTAA-3' at the 5'-terminus ends of nuclear chromosomes.

[#]rDNA repeats presence

Supplementary Table 7 Characteristics of XN chromosomes

ChrID*	Size(bp)	GC%	Genes	Repeat%	LTR%	Heterozygosity‰
Chr01	3,358,403	47.95	1101	13.84	10.63	2.22
Chr02	2,895,836	47.86	874	18.13	14.14	2.67
Chr03#	3,532,025	47.42	1105	23.70	18.57	2.48
Chr04	3,756,861	48.25	1239	11.60	8.43	2.50
Chr05	2,163,075	47.53	671	16.30	12.19	2.69
Chr06	2,432,125	47.04	682	20.81	16.58	2.80
Chr07	2,303,118	47.56	706	17.84	13.89	2.40
Chr08	2,132,961	47.76	617	19.78	16.35	2.62
Chr09	2,268,458	47.11	658	31.48	25.05	2.95
Chr10	2,166,229	47.39	605	30.47	25.12	2.90
Chr11	2,099,829	47.21	576	26.62	20.76	2.28
Chr12	1,953,953	47.56	584	25.46	16.36	2.72
Chr13	2,271,624	47.63	664	15.84	12.65	2.28
Chr14#	2,094,219	47.53	588	27.12	21.29	2.87
Chr15	1,669,390	47.13	461	37.30	28.78	2.37
Chr16	3,605,667	48.18	1174	10.77	7.55	2.27

*telomeric sequence 5'-TTAGG-3' at the 3'-terminus and 5'-CCTAA-3' at the 5'-terminus ends of nuclear chromosomes.

#rDNA repeats presence

Supplementary Table 8 tRNA characteristic constituents of *Rhizoctonia* spp.

Amino acid	Codon	Anticodon	JNa*	JNb*	LY	XN
Ala	GCU	AGC	4	1	4	5
Ala	GCC	GGC	0	0	0	0
Ala	GCA	UGC	2	2	2	3
Ala	GCG	CGC	2	2	2	2
Arg	CGU	ACG	4	4	4	6
Arg	CGC	GCG	0	0	0	0
Arg	CGA	UCG	2	2	2	4
Arg	CGG	CCG	1	1	1	1
Arg	AGA	UCU	1	1	1	1
Arg	AGG	CCU	1	1	1	2
Asn	AAU	AUU	0	0	0	0
Asn	AAC	GUU	3	3	3	4
Asp	GAU	AUC	0	0	0	0
Asp	GAC	GUC	5	5	5	6
Cys	UGU	ACA	0	0	0	0
Cys	UGC	GCA	2	2	2	2
Gln	CAA	UUG	2	2	2	2
Gln	CAG	CUG	3	3	3	3
Glu	GAA	UUC	2	2	2	6
Glu	GAG	CUC	5	5	5	5
Gly	GGU	ACC	0	0	0	0
Gly	GGC	GCC	5	5	6	8
Gly	GGA	UCC	3	3	3	5
Gly	GGG	CCC	2	2	2	2
His	CAU	AUG	0	0	0	0
His	CAC	GUG	3	3	3	3
Ile	AUU	AAU	5	5	5	6
Ile	AUC	GAU	0	0	0	0
Ile	AUA	UAU	1	1	1	1
Leu	UUA	UAA	1	1	1	1
Leu	UUG	CAA	2	2	2	3
Leu	CUU	AAG	3	2	3	4
Leu	CUC	GAG	0	0	0	0
Leu	CUA	UAG	1	1	1	1
Leu	CUG	CAG	2	1	2	2
Lys	AAA	UUU	2	2	2	4
Lys	AAG	CUU	6	5	6	6
Met	AUG	CAU	4	4	4	7
Phe	UUU	AAA	0	0	0	0
Phe	UUC	GAA	3	3	3	4
Pro	CCU	AGG	2	2	2	1
Pro	CCC	GGG	0	0	0	0
Pro	CCA	UGG	2	2	2	3
Pro	CCG	CGG	2	2	2	2
Ser	UCU	AGA	2	1	2	5
Ser	UCC	GGA	0	0	0	0
Ser	UCA	UGA	1	1	1	1
Ser	UCG	CGA	2	2	2	2
Ser	AGU	ACU	0	0	0	0
Ser	AGC	GCU	2	2	2	4
Thr	ACU	AGU	3	3	3	4
Thr	ACC	GGU	0	0	0	0
Thr	ACA	UGU	1	2	1	4
Thr	ACG	CGU	2	2	2	2
Trp	UGG	CCA	2	2	2	2
Tyr	UAU	AUA	0	0	0	0
Tyr	UAC	GUA	3	3	3	3
Val	GUU	AAC	4	5	4	5
Val	GUC	GAC	0	0	0	0
Val	GUA	UAC	1	1	1	2
Val	GUG	CAC	2	2	2	2
Stop	UAA	UUA	0	0	0	0
Stop	UAG	CUA	0	0	0	0
Stop	UGA	UCA	0	0	0	0
Total	/	/	113	108	114	151

*subgenomes of JNa and JNb were divided from JN genome.

Supplementary Table 9 Gene numbers predicted by different methods

Gene prediction	JN	LY	XN
Augustus	29,104	15,066	12,884
GeneMark-ES	29,302	15,107	13,122
RNA-Seq	23,598	12,908	11,023
Exonerate	30,047	14,893	13,407
EVM final set*	29,028	14,549	12,349

*The EVM (EVidenceModeler) software combines ab intio gene predictions and protein and transcript alignments into weighted consensus gene structures.

Supplementary Table 10 Characteristics of genes predicted from different *Rhizoctonia* spp.

Strain	No. genes	Gene size (bp)	Gene density (per Mb)	Exons per gene	Exon size (bp)	Introns per gene	Intron size (bp)
JN[#]	29,028	1,895	301	6.25	250	5.25	64
LY[#]	14,549	1,863	299	6.18	247	5.18	66
XN[#]	12,349	2,110	300	6.95	233	5.95	82
AG1-1A[a]	10,489	1,628	284	5.78	213	4.78	84
AG1-IB[b]	12,616	1,788	294	6.26	218	5.26	78
AG3[c]	12,720	1,752	246	6.47	214	5.47	67
AG8[d]	13,420	1,209	350	4.93	192	3.93	66
AG2-2IIIB[e]	11,897	2,245	212	6.68	221	5.68	71

[a]*R. solani* AG1-IA GD118 (Zheng et al., 2013) [2]

[b]*R. solani* AG1-IB 7/3/14 (Wibberg et al., 2013) [3]

[c]*R. solani* AG3 Rhs1AP (Cubeta et al., 2014) [4]

[d]*R. solani* AG8 WAC10335 (Hane et al., 2014) [5]

[e]*R. solani* AG2-2IIIB BBA69670 (Wibberg et al., 2016) [6]

[#]The student's t-test was performed among JN, LY, and XN genomic data and the results were below.

P-value	gene size	exons per gene	introns per gene	exon size	intron size
JN-LY	0.0984	0.0821	0.0821	0.0691	0.0877
JN-XN	1.26e-36	1.64e-31	1.64e-31	3.90e-06	9.04e-132
LY-XN	1.07e-39	6.98e-31	6.98e-31	3.15e-11	5.29e-108

Supplementary Table 11 KOG function classification of *Rhizoctonia* spp.

KOG family	JN	LY	XN
Information storage and processing	3556	1802	1625
[J] Translation, ribosomal structure and biogenesis	740	373	341
[A] RNA processing and modification	1001	507	470
[K] Transcription	801	391	397
[L] Replication, recombination and repair	717	364	293
[B] Chromatin structure and dynamics	297	167	124
Cellular processes and signaling	5820	2772	2397
[D] Cell cycle control, cell division, chromosome partitioning	311	150	124
[Y] Nuclear structure	65	34	31
[V] Defense mechanisms	339	162	139
[T] Signal transduction mechanisms	1295	615	569
[M] Cell wall/membrane/envelope biogenesis	618	295	190
[N] Cell motility	17	8	8
[Z] Cytoskeleton	412	198	148
[W] Extracellular structures	67	29	21
[U] Intracellular trafficking, secretion, and vesicular transport	668	333	306
[O] Posttranslational modification, protein turnover, chaperones	2028	948	861
Metabolism	6405	3202	2918
[C] Energy production and conversion	443	217	222
[G] Carbohydrate transport and metabolism	1717	866	840
[E] Amino acid transport and metabolism	392	197	203
[F] Nucleotide transport and metabolism	275	141	122
[H] Coenzyme transport and metabolism	100	53	50
[I] Lipid transport and metabolism	657	303	242
[P] Inorganic ion transport and metabolism	449	244	189
[Q] Secondary metabolites biosynthesis, transport and catabolism	2372	1181	1050
Poorly characterized	13247	6773	5409
[R] General function prediction only	3921	1911	2361
[S] Function unknown	9326	4862	3048
Total	29028	14549	12349

Supplementary Table 12 Gene ortholog groups in JN and LY genomes

Term		LY		
		Single-copy	Two-copy	**Total**
JN	Single-copy	1912	44	**1956**
	Two-copy	7812	107*	**7919**
	**Total**	**9724**	**151**	**9875**

*Numbers shown here were numbers of gene ortholog groups (e.g., 107 two-copy ortholog groups contain 214 genes).

Supplementary Table 13 Proportion of repeat sequence contents in *Rhizoctonia* isolates

Repeat class	%for: Length of classified repeats				
	JN[*]	JNa	JNb	LY	XN
**DNA transposon**	2.40	2.62	2.17	2.56	1.68
CMC-EnSpm	0.82	0.89	0.74	0.70	1.12
TcMar-Tc1	0.83	0.67	1.00	0.40	0.54
hAT	0.76	1.07	0.43	1.46	0.03
**LINE**	1.60	1.60	1.59	1.22	1.38
CR1	0.32	0.38	0.26	0.07	0.09
Jockey	0.02	0.01	0.02	0.04	0.23
L1	0.53	0.53	0.53	0.94	0.26
R1	0.44	0.48	0.41	0.10	0.29
Tad1	0.29	0.20	0.38	0.07	0.50
**LTR**	12.51	15.02	9.89	12.76	16.07
Copia	1.00	1.00	1.00	1.44	1.79
ERV1	0.32	0.41	0.23	0.20	0.28
Gypsy	10.59	13.00	8.06	10.13	13.76
other	0.60	0.61	0.59	0.99	0.23
Other	3.39	3.56	3.21	4.34	1.69
Low_complexity	0.04	0.04	0.04	0.04	0.06
Simple_repeat	0.22	0.23	0.22	0.24	0.29
Unknown	3.13	3.29	2.96	4.07	1.35
**Total**	**19.90**	**22.81**	**16.86**	**20.88**	**20.82**

[*]Divided into JNa and JNb subgenomes.

Abbreviations: hAT, hobo-Ac-Tam transposon; LINE, long interspersed nuclear elements; LTR, long terminal repeat-retrotransposons; ERV1, endogenous retrovirus.

Supplementary Table 14 Comparison of the repetitive elements between Illumina and PacBio sequences assembled genomes

Genomes	Illumina genomes					PacBio genomes				
	DNA	LTR	Non-LTR	Other	**Total**	DNA	LTR	Non-LTR	Other	**Total**
JN*	2.50	9.56	1.10	3.96	**17.12**	2.40	12.51	1.60	3.39	**19.90**
JNa	2.67	9.32	1.00	4.15	**17.14**	2.62	15.02	1.60	3.56	**22.81**
JNb	2.19	9.17	1.16	3.55	**16.08**	2.17	9.89	1.59	3.21	**16.86**
LY	2.02	9.58	1.31	3.80	**16.72**	2.56	12.76	1.22	4.34	**20.88**
XN	1.13	14.47	1.14	1.70	**18.44**	1.68	16.07	1.38	1.69	**20.82**

*Divided into JNa and JNb subgenomes.

Supplementary Table 15 Summary of the full-length LTRs (FL-RTs) in *Rhizoctonia* spp.

Type	Quantity	Average length/bp	Min length/bp	Max length/bp	Size/bp	%Genome
JN*	428	9,124	4,991	21,320	3,905,263	4.05
JNa	276	9,157	4,991	21,320	2,527,378	5.09
JNb	152	9,065	5,003	20,476	1,377,885	2.95
LY	213	9,139	4,987	21,313	1,946,654	4.00
XN	291	7,075	6,424	20,205	2,058,871	5.01

*Divided into JNa and JNb subgenomes.

Supplementary Table 16 Distribution of SNPs and InDels in JN subgenomes

Term	Sub	Type	Genomic					Syntenic
			Intron	Exon	Repeat	Intergenic	Total	Exon
SNP	**JNa**	Test regions (kbp)	4,491	18,818	9,267	14,876	47,451	16,286
		SNP (bp)	30,715	67,381	5,834	76,941	180,871	57,152
		SNP (‰)	6.84	3.58	0.63	5.17	3.81	3.51
		Ka	/	0.0037	/	/	/	0.0033
		Ks	/	0.0168	/	/	/	0.0160
		Ka/Ks[#]	/	0.2212	/	/	/	0.2062
	**JNb**	Test regions (kbp)	3,857	15,630	1,646	9,842	30,976	13,395
		SNP (bp)	278,008	603,196	22,238	569,632	1,473,074	512,059
		SNP (‰)	72.07	38.59	13.51	57.87	47.56	38.23
		Ka	/	0.0181	/	/	/	0.0164
		Ks	/	0.1053	/	/	/	0.1060
		Ka/Ks[#]	/	0.1722	/	/	/	0.1550
		SNP (‰)	72.07	38.59	13.51	57.87	47.56	38.23
InDel	**JNa**	Test regions (kbp)	4,491	18,818	9,267	14,876	47,451	16,286
		InDel No.	1,245	818	534	3,440	6,037	459
		InDel (‰)	0.28	0.04	0.06	0.23	0.13	0.03
	**JNb**	Test regions (kbp)	3,857	15,630	1,646	9,842	30,976	13,395
		InDel No.	5,311	949	506	10,289	17,055	563
		InDel (‰)	1.38	0.06	0.31	1.05	0.55	0.04

[#]Ratio of nonsynonymous to synonymous substitutions (*Ka/Ks*) in the SNPs of exonic regions was relative higher in JNa than JNb estimated from the whole subgenome (P = 3.9e-21) or the homeologous regions (P = 5.1e-32). P-values were calculated using student's t-test.

Supplementary Table 17 Distribution of heterozygous SNP (hSNP) in *Rhizoctonia* spp.

Isolates	Type	Intronic	Exonic	repetitive	Intergenic	Genome
**JNa***	regions tested (bp)	4,351,863	20,280,978	11,689,939	13,242,291	49,565,071
	hSNP (bp)	1,445	5,265	3,199	926	10,835
	hSNP (‰)	0.33	0.26	0.27	0.07	0.22
**JNb***	regions tested (bp)	4,472,631	19,969,089	9,402,180	12,881,550	46,725,450
	hSNP (bp)	1,110	4,404	699	1,345	7,558
	hSNP (‰)	0.25	0.22	0.07	0.10	0.16
**LY**	regions tested (bp)	4,740,970	19,477,272	10,852,097	13,585,502	48,655,841
	hSNP (bp)	3,148	9,149	10,164	8,496	30,957
	hSNP (‰)	0.66	0.47	0.94	0.63	0.64
**XN**	regions tested (bp)	5,578,229	16,738,923	11,295,531	7,491,377	41,104,060
	hSNP (bp)	16,213	40,022	32,648	16,471	105,354
	hSNP (‰)	2.91	2.39	2.89	2.20	2.56

*subgenomes of JNa and JNb were divided from JN genome.

Supplementary Table 18 Category of gene loss and gain in JN subgenomes

KOG	JNa-loss	JNb			
		-loss	-insertion	-substituted	-substitution
**Information storage and processing**	**41**	**130**	**62**	**112**	**166**
[A] RNA processing and modification	5	33	15	28	26
[B] Chromatin structure and dynamics	6	10	0	5	3
[J] Translation, ribosomal structure and biogenesis	10	19	6	18	32
[K] Transcription	10	24	10	34	58
[L] Replication, recombination and repair	10	44	31	27	47
**Cellular processes and signaling**	**57**	**162**	**115**	**195**	**360**
[D] Cell cycle control, cell division, chromosome partitioning	0	9	2	8	18
[M] Cell wall/membrane/envelope biogenesis	7	22	14	27	44
[N] Cell motility	0	1	0	0	0
[O] Posttranslational modification, protein turnover, chaperones	20	59	37	59	121
[T] Signal transduction mechanisms	18	33	36	57	103
[U] Intracellular trafficking, secretion, and vesicular transport	2	12	3	12	15
[V] Defense mechanisms	4	12	7	12	27
[W] Extracellular structures	2	0	1	1	4
[Y] Nuclear structure	0	4	1	1	3
[Z] Cytoskeleton	4	10	14	18	25
**Metabolism**	**77**	**209**	**66**	**183**	**237**
[C] Energy production and conversion	4	14	1	12	14
[E] Amino acid transport and metabolism	4	16	1	7	9
[F] Nucleotide transport and metabolism	2	8	1	6	3
[G] Carbohydrate transport and metabolism	24	30	16	54	56
[H] Coenzyme transport and metabolism	2	4	0	1	2
[I] Lipid transport and metabolism	5	15	7	18	34
[P] Inorganic ion transport and metabolism	3	28	4	20	12
[Q] Secondary metabolites biosynthesis, transport and catabolism	33	94	36	65	107
**Poorly characterized**	**166**	**854**	**661**	**1122**	**1388**
[R] General function prediction only	33	263	272	412	494
[S] Function unknown	133	591	389	710	894
**Total**	**341**	**1355**	**904**	**1612**	**2151**

Supplementary Table 19 Gene loss detected at chromosome ends of JN subgenomes

ChrID	5'			3'		
	gene loss	Transposase	TE nearby the breakage site (≤ 5 kb)	gene loss	Transposase	TE nearby the breakage site (≤ 5 kb)
A15	32	5	Yes	0	0	No
B01	0	0	No	15	4	Yes
B02	0	0	No	24	2	Yes
B03	0	0	No	3	0	No
B04	0	0	No	2	1	Yes
B05	0	0	No	15	5	Yes
B06	0	0	No	38	7	Yes
B07	0	0	No	25	4	Yes
B08	11	1	Yes	91	7	Yes
B09	0	0	No	2	0	No
B10	0	0	No	419	40	Yes
B11	12	3	Yes	5	2	Yes
B12	23	8	Yes	1	0	No
B13	30	3	Yes	0	0	No
B14	0	0	No	41	5	Yes
B15	0	0	No	0	0	No
B16	16	0	No	18	4	Yes

Supplementary Table 20 List of gene exchanges between JN subgenomes

ChrID	single gene exchange	multiple gene exchange
Chr01	6	1
Chr02	11	2
Chr03	11	0
Chr04	20	12
Chr05	7	0
Chr06	6	1
Chr07	7	0
Chr08	10	0
Chr09	7	0
Chr10	1	0
Chr11	4	0
Chr12	2	0
Chr13	41	15
Chr14	39	12
Chr15	12	3
Chr16	15	0
**total**	**199**	**46**

Supplementary Table 21 KOG function classification of Decelerated/Decelerated and Accelerated/Accelerated homeologs in JN subgenomes

KOG family	Accelerated/ Accelerated	Decelerated/ Decelerated
**Information storage and processing**	**7**	**21**
[J] Translation, ribosomal structure and biogenesis	1	5
[A] RNA processing and modification	4	8
[K] Transcription	2	5
[L] Replication, recombination and repair	0	2
[B] Chromatin structure and dynamics	0	1
**Cellular processes and signaling**	**17**	**20**
[D] Cell cycle control, cell division, chromosome partitioning	1	2
[Y] Nuclear structure	0	0
[V] Defense mechanisms	3	2
[T] Signal transduction mechanisms	5	6
[M] Cell wall/membrane/envelope biogenesis	1	1
[N] Cell motility	0	0
[Z] Cytoskeleton	0	1
[W] Extracellular structures	0	0
[U] Intracellular trafficking, secretion, and vesicular transport	6	5
[O] Posttranslational modification, protein turnover, chaperones	1	3
**Metabolism**	**13**	**22**
[C] Energy production and conversion	0	4
[G] Carbohydrate transport and metabolism	2	9
[E] Amino acid transport and metabolism	2	1
[F] Nucleotide transport and metabolism	2	0
[H] Coenzyme transport and metabolism	2	1
[I] Lipid transport and metabolism	1	2
[P] Inorganic ion transport and metabolism	2	3
[Q] Secondary metabolites biosynthesis, transport and catabolism	2	2
**Poorly characterized**	**13**	**23**
[R] General function prediction only	3	7
[S] Function unknown	10	16
**Total**	**50**	**86**

Supplementary Table 22 Prediction of secreted and effector proteins in *Rhizoctonia* spp.

Function	Secreted protein				Effector protein			
	JNa*	JNb*	LY	XN	JNa*	JNb*	LY	XN
aspartyl protease	22	23	21	17	3	4	2	3
carbohydrate esterase family protein	26	27	24	24	8	9	9	8
carbohydrate-binding domain protein	8	8	7	3	7	7	5	3
cellulase	16	14	16	14	4	5	4	5
CFEM domain protein[1]	9	15	12	3	2	3	3	2
chitin deacetylase	12	13	11	12	6	6	5	5
chitinase	7	7	5	5	0	0	0	1
cutinase	2	3	2	4	3	3	3	3
cysteine-rich secretory family protein	8	7	7	4	7	6	7	3
endo-beta-1,4-xylanase	13	15	13	7	5	6	6	0
glycoside hydrolase family protein	125	126	119	95	42	39	40	29
lipase	12	10	11	11	8	7	7	9
LysM domain protein	3	2	2	3	1	1	1	1
lysozyme	2	3	1	1	1	1	0	0
lytic polysaccharide mono-oxygenase	2	1	2	3	1	1	2	3
pathogenesis-related protein PR5	11	10	10	11	12	13	11	12
pectate lyase	14	15	14	57	5	5	6	42
pectinesterase	7	6	6	5	2	1	2	0
peptidyl-Lys metalloendopeptidase	18	11	17	14	15	11	13	10
peroxidase family protein	3	3	2	4	1	0	0	1
polygalacturonase	7	8	9	18	5	5	5	11
polysaccharide lyase family protein	8	6	6	9	2	4	4	2
rhamnogalacturonate lyase	4	4	3	8	/	/	/	/
ricin-type beta-trefoil lectin domain protein	2	2	2	1	2	0	2	1
subtilisin-like protease 8	3	2	3	5	0	0	0	1
trehalase	3	5	3	2	/	/	/	/
tyrosinase	15	15	14	21	13	13	11	18
WSC domain protein[2]	3	1	2	3	/	/	/	/
Other function protein	213	231	208	178	33	34	30	25
Unknown function protein	328	316	324	227	211	200	215	156
**Total**	**906**	**909**	**876**	**769**	**399**	**384**	**393**	**354**

*subgenomes of JNa and JNb were divided from JN genome.

[1]CFEM, cysteine-rich fungal extracellular membrane.

[2]WSC, cell wall integrity and stress response component.

Supplementary References

1. Cuomo, C.A. et al. Comparative analysis highlights variable genome content of wheat rusts and divergence of the mating loci. *G3 (Bethesda)* **7**, 361-376 (2017).
2. Zheng, A. et al. The evolution and pathogenic mechanisms of the rice sheath blight pathogen. *Nat. Commun.* **4**, 1424 (2013).
3. Wibberg, D. et al. Establishment and interpretation of the genome sequence of the phytopathogenic fungus *Rhizoctonia solani* AG1-IB isolate 7/3/14. *J Biotechnol* **167**, 142-155 (2013).
4. Cubeta, M.A. et al. Draft genome sequence of the plant-pathogenic soil fungus *Rhizoctonia solani* anastomosis group 3 strain Rhs1AP. *Genome Announc.* **2,** e01072-14 (2014).
5. Hane, J.K., Anderson, J.P., Williams, A.H., Sperschneider, J. & Singh, K.B. Genome sequencing and comparative genomics of the broad host-range pathogen *Rhizoctonia solani* AG8. *PLoS Genet* **10**, e1004281 (2014).
6. Wibberg, D. et al. Genome analysis of the sugar beet pathogen *Rhizoctonia solani* AG2-2IIIB revealed high numbers in secreted proteins and cell wall degrading enzymes. *BMC Genomics* **17**, 245 (2016).
